# Antithrombotic Polymers: A Narrative Review on Current and Future Strategies for Their Design, Synthesis, and Application

**DOI:** 10.3390/ijms27021026

**Published:** 2026-01-20

**Authors:** Anna Smola-Dmochowska, Natalia Śmigiel-Gac, Katarzyna Jelonek, Kamila Lewicka-Brzoza, Jakub Bojdol, Piotr Dobrzyński

**Affiliations:** 1 Centre of Polymer and Carbon Materials, Polish Academy of Sciences, 41-819 Zabrze, Poland; asmola@cmpw-pan.pl (A.S.-D.); ngac@cmpw-pan.pl (N.Ś.-G.); kjelonek@cmpw-pan.pl (K.J.); 2 Faculty of Science and Technology, Jan Dlugosz University in Czestochowa, 13/15 Armii Krajowej Av., 42-200 Czestochowa, Poland; k.lewicka@ujd.edu.pl; 3 Faculty of Medical Sciences in Katowice, Medical University of Silesia, 40-055 Katowice, Poland; jakubbojdol12@gmail.com

**Keywords:** antithrombotic polymers, hemocompatibility, antithrombotic surfaces, drug-delivery strategies, thrombosis prevention, biomimetic polymers, clinical trial

## Abstract

Bleeding and thromboembolism are among the leading causes of mortality worldwide. Thrombosis encompasses both arterial forms—primarily associated with atherosclerosis and leading to heart attacks or strokes—and venous forms. Microvascular thrombosis typically arises in the context of sepsis or systemic inflammation, and it became particularly prominent during the COVID-19 pandemic, substantially contributing to increased mortality. Given this burden, the rapid development of new therapies using advanced techniques and materials to prevent and treat these conditions is essential. This review summarizes recent advances in the design of antithrombotic polymers, discussing mechanisms of action, surface-modification strategies, and current clinical and preclinical applications. It also outlines criteria for evaluating hemocompatibility, describes *in vitro* and *in vivo* testing methods, and highlights key barriers to translating these materials into clinical practice. The review concludes by identifying promising directions for future research, including multifunctional approaches that combine antifouling properties, controlled drug release, and bioresistance strategies with the greatest potential to reduce thromboembolic complications associated with medical materials. It further evaluates the progress made to date in combating thrombotic diseases and identifies remaining gaps in the development and clinical implementation of new antithrombotic materials.

## 1. Introduction 

**Figure 1:**
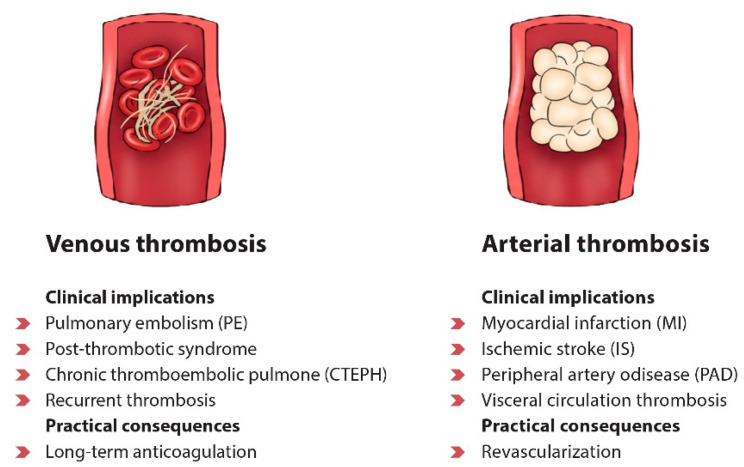
Pathophysiological and Clinical Differences Between Venous and Arterial Thrombosis.

Bleeding and thromboembolic conditions are significant causes of death and serious health issues around the world. In 2010, it was estimated that thromboembolic conditions accounted for up to one in four deaths, and they continue to be one of the leading causes of mortality. Thrombosis, as a disease category, includes both arterial and venous thromboses (see Figure 1). Arterial thrombosis, often linked with atherosclerosis, can result in myocardial infarction, peripheral artery disease, and ischemic stroke. Deep vein thrombosis (DVT) and pulmonary embolism (PE) are the primary complications associated with venous thromboembolism (VTE). Additionally, microvascular thrombosis typically occurs in the context of sepsis or sterile inflammation. This phenomenon was particularly evident during COVID-19, where thromboembolic complications significantly contributed to mortality [1,2,3,4].

Venous thromboembolism, encompassing deep vein thrombosis and pulmonary embolism, ranks as the third most common cardiovascular condition after myocardial infarction and stroke. Its annual incidence is estimated at 1–2 cases per 1000 individuals. VTE represents a major global health issue, associated with substantial morbidity and mortality, while PE is an independent predictor of reduced survival. The likelihood of recurrence is greatest during the first 6–12 months following the initial event, although it remains elevated over the long term. Anticoagulant therapy is the cornerstone of both prevention and treatment, with careful consideration required to balance the risk of recurrence against bleeding complications. Under well-managed anticoagulation, the recurrence rate is estimated to be around 1–3% depending on population, duration, and whether the cohort includes high-risk groups (cancer, antiphospholipid syndrome, obesity, pregnancy, etc.) [5].

In transplantology, problems with blood clots can lead to serious health complications later on. Blood-contacting medical devices (BCMDs) have revolutionized modern medicine and contributed to saving millions of lives. These devices include artificial hearts, prosthetic valves, pacemakers, stents, central venous catheters, and external components of extracorporeal systems, such as cardiopulmonary bypass, extracorporeal membrane oxygenation (ECMO), and dialysis circuits. Despite their widespread clinical use, BCMD remain susceptible to adverse patient outcomes due to shear-induced blood cell damage and device-related thrombosis. These complications may lead to potentially life-threatening events, such as device failure, ischemic or embolic stroke, hemolytic anemia, and even acute kidney injury. Although advances in medical technology have improved the performance of BCMD, they continue to face significant challenges stemming from the complex interplay between the human body, blood components, and synthetic materials. When blood comes into contact with artificial surfaces, it triggers a cascade of biochemical reactions that can disrupt the coagulation system, resulting in both thrombotic and hemorrhagic events. Thrombosis poses a serious risk not only to device functionality but also to overall patient safety. As more patients rely on blood-contacting medical devices, reducing the risks they pose is key to lowering complications and improving survival rates [6,7,8,9].

To mitigate the risks of thrombosis, inflammation, and infection associated with the use of blood-contacting medical devices, systemic pharmacotherapy with anticoagulants, mainly heparin and antibiotics, remains a common clinical strategy. However, this approach is often accompanied by serious complications, including a high risk of hemorrhage and heparin-induced thrombocytopenia. Clinical and pharmacovigilance data indicate that a significant proportion of deaths related to adverse drug reactions are due to the use of systemic anticoagulants and antibiotics [6]. Systemically administered antibiotics may cause serious adverse reactions such as toxic epidermal necrolysis, drug fever, thrombophlebitis, or hypersensitivity syndromes. Typical causes of thrombosis treatment failure are non-compliance or subtherapeutic exposure—missed dose, incorrect dosing, malabsorption syndrome, vomiting/diarrhea, or poor oral medication availability [10,11,12]. Another factor contributing to thrombosis treatment failure is drug–drug or drug-food interaction [13,14]. It is necessary to closely monitor the intensity of the anticoagulant effect using the international normalized ratio (INR) [13]. An important factor in the success of thrombosis treatment is the issue of drug resistance, i.e., a reduced response to a given dose of a therapeutic agent, which may occur earlier or be caused by prior drug exposure [15]. The term “heparin resistance” has gained importance and become more noticeable in the context of the COVID-19 pandemic, as many patients required higher than expected doses of heparin as part of their COVID-19 treatment or if they required extracorporeal membrane oxygenation (ECMO) [16].

Despite the remarkable progress achieved in anticoagulant therapy over the past decades, current approaches remain suboptimal. This limitation primarily stems from an incomplete elucidation of the intricate structural and functional characteristics of platelets, whose multifaceted roles in hemostasis and thrombosis continue to challenge comprehensive therapeutic targeting [17]. This has highlighted an urgent clinical need for strategies that minimize complications while reducing or eliminating systemic drug delivery. In response, surface functionalization technologies have been developed to address thrombosis, inflammation, and infection directly at the interface of blood-contacting devices. In this context, there is a growing demand for materials that are not only mechanically and biologically compatible with the body but also actively counteract the initiation and progression of clotting. Polymers occupy a unique position in this field. Unlike inorganic coatings (e.g., metal oxides) or simple pharmacological carriers, they offer a wide range of chemical and physicochemical engineering possibilities—from surface modification and macromolecular structure to functionalization with bioactive groups. Their modular structure allows for precise adjustment of properties (hydrophobicity/hydrophilicity, surface charge, and the ability to release active molecules). Compared to traditional pharmacological treatments, the targeted use of polymers enables local action, reducing the risk of systemic side effects (e.g., bleeding during anticoagulant therapy). Compared to inorganic materials or metals, polymers also exhibit improved mechanical flexibility and biocompatibility, facilitating their use in flexible vascular prostheses, catheters, and stent coatings. Due to these properties, antithrombotic polymers have become a key focus of biomaterials research. Strategies include both passive modifications (e.g., antifouling coatings that limit protein adsorption) and active modifications (e.g., heparin immobilization, release of nitric oxide donors, or antiplatelet drugs) [18].

## 2. Physiological Basics of Coagulation and Antithrombotic Mechanisms

Effective thrombosis treatment requires understanding hemostasis, a system balancing blood fluidity and clot formation. It involves three key elements: the vascular endothelium, platelets, and the plasma coagulation/fibrinolytic systems [19].

### 2.1. Role of the Vascular Endothelium

**Scheme 1:**
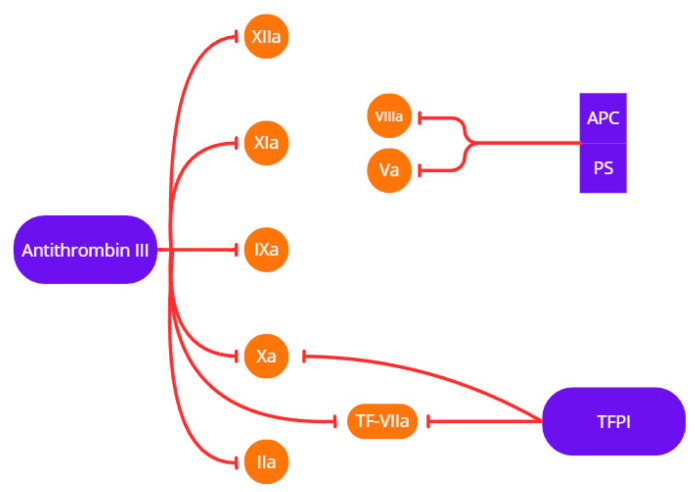
Endogenous anticoagulant pathways: targets of antithrombin III, TFPI, and APC/Protein S.

The endothelium is the primary defense against uncontrolled coagulation. It inhibits platelet activation through its negatively charged surface and the release of prostacyclin (PGI_2_) and nitric oxide (NO). Additionally, it regulates the coagulation cascade via natural inhibitors: Antithrombin III (neutralizing thrombin and factors IXa–XIIa), TFPI (blocking the TF–VIIa/Xa complex), and thrombomodulin. The latter binds thrombin to activate Protein C, which, alongside Protein S, inactivates factors Va and VIIIa to limit clot amplification [20] (see Scheme 1). The endothelium also plays a key role in fibrinolysis. It produces plasminogen activators (t-PA and u-PA), which initiate the conversion of plasminogen to plasmin, the enzyme responsible for degrading cross-linked fibrin and dissolving the clot, helping maintain vessel patency [21,22]. When endothelial injury occurs, anticoagulant mechanisms are lost, and the exposed extracellular matrix—rich in collagen and von Willebrand factor—becomes a site for platelet adhesion and initiation of coagulation.

### 2.2. The Importance of Platelets

Once dismissed as “cellular dust,” platelets are now recognized as vital for hemostasis, angiogenesis, and immunity. They are the cornerstone of both primary and secondary hemostasis, forming the initial plug and providing the surface for fibrin production. This functionality is driven by their granules: α-granules release proteins such as fibrinogen, von Willebrand factor, and cytokines, while δ-granules (dense bodies) secrete small molecules like ADP, serotonin, and calcium ions (Ca^2+^) to amplify aggregation and the coagulation cascade [22]. Platelet activation leads to the exposure of phosphatidylserine on the outer membrane surface, providing a site for prothrombinase complex assembly. The platelet surface is rich in receptors (e.g., P2Y_12_ for ADP, TP for thromboxane A_2_, and the GPIIb/IIIa complex binding fibrinogen), which are targets for numerous antiplatelet drugs (e.g., clopidogrel, prasugrel, ticlopidine) used in the treatment of thrombotic diseases [23].

### 2.3. The Plasma Coagulation System and the Coagulation Cascade

**Scheme 2:**
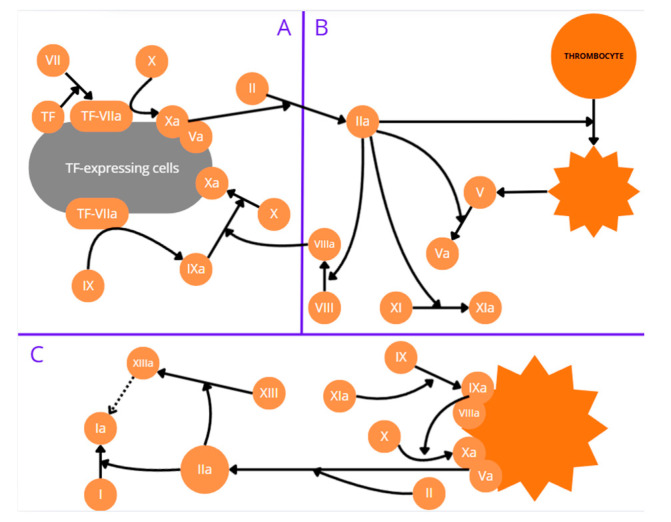
Visual representation of the clotting cascade with the three phases highlighted: (**A**)—initiation; (**B**)—amplification; (**C**)—propagation.

Modern understanding of the coagulation cascade goes beyond the classic intrinsic and extrinsic pathways. Today, it is described as an integrated model in which not only zymogen activation sequences matter, but also the essential role of cellular surfaces that enable localization and concentration of enzymatic reactions [24]. The coagulation process can be divided into three phases: initiation, amplification, and propagation (see Scheme 2). Initiation phase, traditionally described as the extrinsic pathway, begins with endothelial injury exposing the extracellular matrix along with tissue factor (TF), the key initiator of the coagulation cascade. TF binds factor VII, leading to its activation. The TF–FVIIa complex participates in generating FIXa and FXa. FXa then associates with its cofactor FVa to form the prothrombinase complex on the surface of TF-expressing cells. Prothrombinase catalyzes the conversion of prothrombin (FII) to thrombin (FIIa). The amount of thrombin produced at this stage is insufficient to form a stable clot, but in the amplification phase, it activates platelets that adhere to the site of injury and release FV, which is then converted to FVa by thrombin. Thrombin also activates FVIII, the cofactor for FIXa, increasing FXa generation, and additionally activates FXI. These events intensify the coagulation cascade. Propagation phase, FXIa activates FIX, which binds thrombin-activated FVIIIa to form the tenase complex that activates FX to FXa. The FXa/FVa complex then generates sufficient thrombin to produce enough fibrin to form a stable clot. In the final step, thrombin activates FXIII, which catalyzes covalent cross-linking of adjacent fibrin strands, forming a stable cross-linked fibrin clot [19].

### 2.4. The Process of Fibrinolysis

**Scheme 3:**
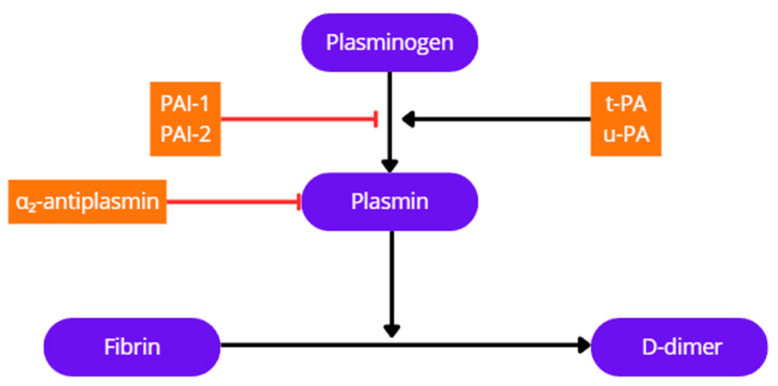
Regulation of fibrinolysis: plasminogen activation and plasmin inhibition.

Fibrinolysis is a highly regulated process where plasmin, generated from plasminogen by t-PA or u-PA, degrades fibrin. The short half-life of these activators (4–8 min) is maintained by inhibitors PAI-1 and PAI-2, while α_2_-antiplasmin directly inactivates plasmin (Scheme 3). The resulting D-dimer fragments, produced during fibrin cleavage, serve as essential clinical markers for diagnosing and monitoring conditions such as DIC, pulmonary embolism, and deep vein thrombosis (DVT) [25].

After blood comes into contact with the surface of a polymeric material, the first and key event is the immediate adsorption of plasma proteins. Mainly fibrinogen, albumin, and von Willebrand factor (vWF) deposit on the surface, forming a dynamic “protein corona” [26,27,28]. Upon interaction with the foreign surface, some of these proteins undergo conformational changes, exposing adhesive epitopes that are not accessible in solution. It is this modified protein layer, rather than the material itself, that constitutes the direct interface for blood platelets [29].

Platelets recognize the exposed adhesive motifs (including those in fibrinogen and vWF), adhere to the surface, and subsequently become activated. Activated platelets change their shape, release granular mediators (including procoagulant and pro-inflammatory factors), and form a network of interconnections via fibrinogen bridges [30]. The released factors promote local activation of the coagulation cascade—prothrombin is converted to thrombin (II → IIa), followed by the conversion of fibrinogen into insoluble fibrin [25]. The developing fibrin network stabilizes the platelet aggregate, leading to the formation of a mature hemostatic plug. As a result, a material that initially only came into contact with blood becomes the core of a thrombus, and its surface properties (chemical composition, charge, roughness, hydrophilicity) critically determine the rate and extent of the entire process [31].

**Scheme 4:**
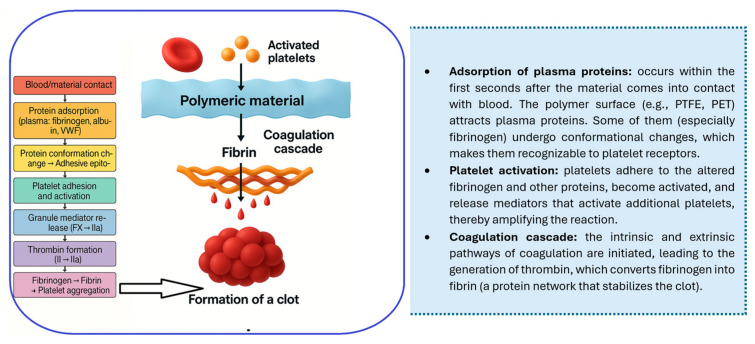
Sequence of events at the blood–polymer interface: immediate adsorption and conformational rearrangement of plasma proteins on the polymer surface, followed by platelet adhesion and activation, release of platelet mediators, activation of the coagulation cascade, and final fibrin formation leading to clot development.

The surface modulates the entire clot lifecycle, from initiation to dissolution. Materials that promote rapid re-endothelialization and limit local thrombin generation maintain a physiological balance with the fibrinolytic system (t-PA plasmin). This synergy reduces the risk of persistent or calcified thrombi forming on implants [32,33] (see Scheme 4).

### 2.5. Therapeutic Goals: Anticoagulation, Antiaggregation, Thrombolysis

The issue of thrombus formation on blood-contacting device surfaces is a persistent complication and the main reason for the failure. The standard procedure to minimize foreign surface-mediated thrombosis is the administration of systemic anticoagulant drugs, direct thrombin inhibitors, and platelet inhibitory agents [34]. Antiplatelet and anticoagulant drugs exert their preventative effects by inhibiting blood clot formation via direct and indirect mechanisms. Following vascular injury or in response to pathological vascular events, platelets are activated and aggregate within blood vessels, commonly forming arterial thrombi [35]. Platelet activation and aggregation are mediated by multiple signaling pathways that serve as key targets for pharmacological intervention. The use of a combination of antiplatelet agents offers additive benefits by modulating multiple pathways, thereby enhancing therapeutic efficacy [36].

**Figure 2:**
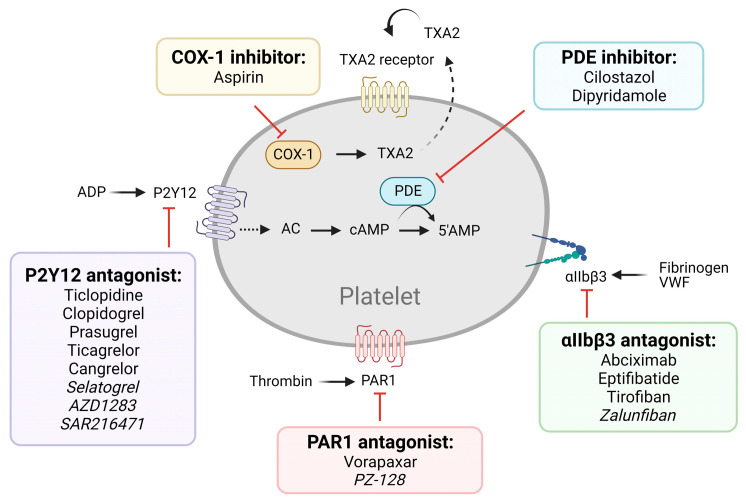
Schematic presentation of commonly used (regular font) and next-generation (italicized) antiplatelet therapy [35].

Antiplatelet therapy is a cornerstone of cardiovascular treatment, reducing thrombotic risk by inhibiting platelet aggregation [37]. Major classes include inhibitors of COX, P2Y12, PDE, PAR1, and glycoprotein IIb/IIIa (Figure 2, Table 1). Aspirin, a primary agent, irreversibly inactivates COX-1, blocking the synthesis of thromboxane (TX)A_2_ [38,39]. P2Y12 inhibitors, which block ADP-mediated activation, are essential for managing ACS and post-PCI patients, typically as part of dual antiplatelet therapy [40,41]. These agents are categorized into thienopyridine prodrugs requiring hepatic bioactivation (e.g., clopidogrel, prasugrel) and direct-acting inhibitors (e.g., ticagrelor, cangrelor) [36].

Genetic variability in CYP450 metabolism results in ~30% of patients being poor or intermediate responders to clopidogrel [42]. Consequently, more potent and predictable agents like ticagrelor and cangrelor are increasingly preferred [43]. Other therapeutic strategies include PDE inhibitors, which elevate cAMP/cGMP to suppress αIIbβ3 antagonist activation and platelet secretion [35]. Glycoprotein IIb/IIIa antagonists—such as abciximab (antibody), eptifibatide (disintegrin) [44], and tirofiban (peptidomimetic)—prevent aggregation by blocking the fibrinogen receptor [45,46,47]. Finally, the PAR1 antagonist vorapaxar inhibits thrombin-induced calcium mobilization, though it carries a significant risk of bleeding [48].

Anticoagulants work through the coagulation cascade by inhibiting specific clotting factors to modulate thrombus formation. Vitamin K antagonists (e.g., warfarin) and heparin were the first anticoagulants approved and remain clinically predominant. Warfarin inhibits Factors II, VII, IX, and X, while heparin inactivates thrombin and Factor Xa. However, their broad mechanism of action increases the risk of adverse bleeding events, particularly in patients with compromised hemostasis. Consequently, the development of targeted oral anticoagulants that selectively inhibit specific coagulation factors is observed [37].

Unlike anticoagulants, thrombolytic agents actively dissolve existing clots by converting plasminogen into the enzyme plasmin. These agents are categorized based on their fibrin-specificity:

Fibrin-specific (e.g., alteplase, tenecteplase): Target plasminogen on the clot surface, localized action reduces systemic bleeding and fibrinogen depletion [49,50,51].

Non-fibrin-specific (e.g., streptokinase, urokinase): Act systemically; streptokinase induces an indirect conformational change in plasminogen to initiate fibrinolysis. Clinically, these agents are critical emergency interventions for acute ischemic stroke, myocardial infarction, and pulmonary embolism [52].

However, systemic anticoagulation may not be effective enough in preventing thrombus formation, which leads to device failure, costly device replacement, and significant risk to the patient. The pharmacologic limitations of anticoagulants and thrombolytics include rapid renal clearance, short half-lives, off-target toxicity, and problems with patient compliance regarding dosing. The major adverse events include severe thrombocytopenia, hemorrhage, and death, particularly in small children, cancer patients, and pregnant women. Therefore, biomaterial-controlled delivery applications of these compounds are attractive and gaining interest. Using biomaterials for encapsulation and controlled release of anticoagulants and thrombolytics may improve therapeutic effects by reducing dosing frequency, maintaining therapeutic plasma concentrations, and limiting off-target distribution. Additionally, they can improve patient compliance and minimize post-administration monitoring requirements [37]. Application of biocompatible materials may include formation of nonadhesive surfaces that minimize protein adsorption and thrombus deposition; immobilization of anticoagulants or active release of antithrombotic compounds [34,53].

Despite over 50 years of research and development in improving blood-material interactions, the only truly hemocompatible surface remains the endothelium. Therefore, preventing coagulation via mechanisms similar to those used by the endothelium can improve the safety and efficacy of biomaterial surfaces that contact blood. Endothelial cells express antithrombotic proteins such as tissue factor pathway inhibitor (TFPI), thrombomodulin, endothelial protein C receptor (EPCR), and heparin-like molecules on their surfaces [53].

## 3. Classes of Polymers Used as Anticoagulant Materials

### 3.1. Natural Polymers and Their Modifications

An increasing number of studies highlight the pivotal role of natural polymers in the treatment of various diseases, underscoring their considerable therapeutic potential. In particular, their efficacy in the management of thromboembolic disorders is noteworthy, as they have been shown to mitigate toxic side effects, reduce recurrence rates, and improve patient outcomes. Moreover, natural polymers provide valuable lead compounds that drive modern pharmaceutical discovery and contribute to the development of first-line therapeutic agents [54,55]. Despite these promising findings, the translation of preclinical evidence into clinical applications remains a significant challenge. Although this article presents a narrative synthesis, systematic reviews of preclinical studies remain crucial for identifying the most promising candidates, clarifying mechanisms, and informing the design of future clinical trials.

**Figure 3:**
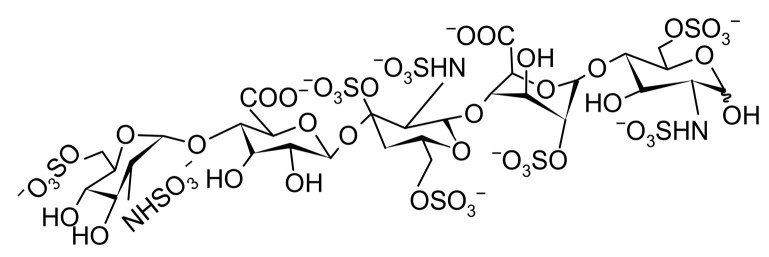
Molecular structure of heparin.

Heparin (Figure 3) is one of the most commonly used anticoagulant macromolecules. Heparin is a natural polymer belonging to the glycosaminoglycan (GAG) group, which plays a key role in regulating blood coagulation.

Structurally, it is a linear polysaccharide composed primarily of repeating disaccharide units containing uronic acid (most commonly iduronic acid) and glucosamine, which is highly sulfated, giving it a high negative charge density. This molecular feature determines the ability of heparin to activate antithrombin III, which then inhibits the activity of thrombin and factor Xa, key elements of the blood coagulation cascade [56,57]. Due to its properties, heparin has found widespread clinical use in the prevention and treatment of thromboembolic diseases, including deep vein thrombosis, pulmonary embolism, and during surgical procedures and dialysis. It is important to emphasize that heparin is one of the oldest and most well-studied natural polymers of pharmacological importance, and its presence in medical practice remains crucial despite the development of newer anticoagulants [58]. Nevertheless, it is important to note that heparin can only be derived from animal tissues, such as porcine intestine or bovine lung, which results in high manufacturing expenses, notable adverse effects, and complicated extraction processes [56]. As a natural product, heparin is susceptible to desulfation, difficult to isolate in large quantities, and carries the risk of pathogen transmission and immunological reactions [59]. Due to its structural heterogeneity, the bioactivity and physiological effects of unfractionated heparin (UFH) are broad and unpredictable. Certain heparin chains interact with plasma proteins, resulting in adverse effects such as osteoporosis from impaired bone metabolism, heparin-induced thrombocytopenia (HIT), and variable anticoagulant responses that require continuous monitoring. The introduction of low molecular weight heparin (LMWH) in the late 1970s and early 1980s allowed for a more predictable activity profile. LMWHs, including enoxaparin, dalteparin, and tinzaparin, are prepared through controlled chemical or enzymatic depolymerization of unfractionated heparin [60]. Although heparin demonstrates good clinical efficacy, its multiple adverse effects—including bleeding, elevated aminotransferase levels, HIT, and allergic reactions—remain a significant concern [61]. Due to difficulties in controlling the anticoagulant effect, research is underway to develop antidotes that reverse the effects of anticoagulants. For a long time, this was practically impossible with heparin. Based on the *in vitro* and *in vivo* studies described by Kałaska and Szczubiałka and their team [62], the heparin-binding copolymer they developed appears to be a promising candidate for neutralizing all clinically relevant low-molecular-weight heparins. However, it is not used clinically. For this reason, intensive research is underway to develop new, safer, and more predictable anticoagulants. Promising avenues include structural modifications of heparin itself and the design of synthetic heparin mimetics, which aim to mimic its biological activity while limiting adverse effects [63]. Although Fondaparinux—a rationally designed heparin mimetic—is currently approved for the prevention and treatment of venous thromboembolism, the search for new anticoagulant heparin mimetics with increased affinity and fewer side effects remains a subject of intense research [64].

Alternatively, molecular simulation strategies have been proposed to mimic heparin by introducing functional sulfate domains into polysaccharides with similar sugar backbones. Hsieh-Wilson’s team, who, using ring-opening metathesis polymerization (ROMP), obtained a series of heparin-based glycopolymers and demonstrated that their structures can be tailored to recapitulate the potent activity of anticoagulant drugs. They discovered that the morphology of the resulting copolymer chains, variations in glycopolymer length, and segments containing sulfonic groups enable modulation of their anticoagulant activity, thus leading to the creation of new drugs with unique, hybrid activities that differ from those of natural and synthetic glycosaminoglycans [65]. 

**Figure 4:**
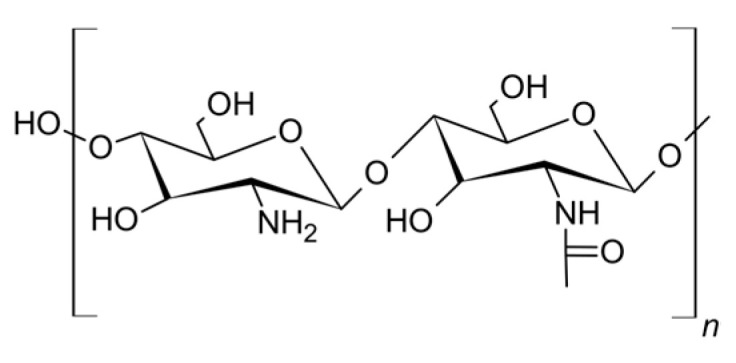
Molecular structure of chitosan.

Chitosan (Figure 4), a linear natural polysaccharide, has proven particularly suitable; when sulfated at specific sites, it acquires heparin-like bioactivity and has found widespread biomedical applications [56,59]. Chitosan is obtained by the deacetylation of chitin, one of the most abundant polysaccharides in nature, extracted from the exoskeleton of crustaceans, squids, or fungi walls. Chitosan is the only naturally positively charged polysaccharide, with abundant free amino groups, which make it soluble in neutral or acidic aqueous media (pH < 6), depending on the degree of deacetylation (DDA), molecular weight, or source [66].

**Figure 5:**
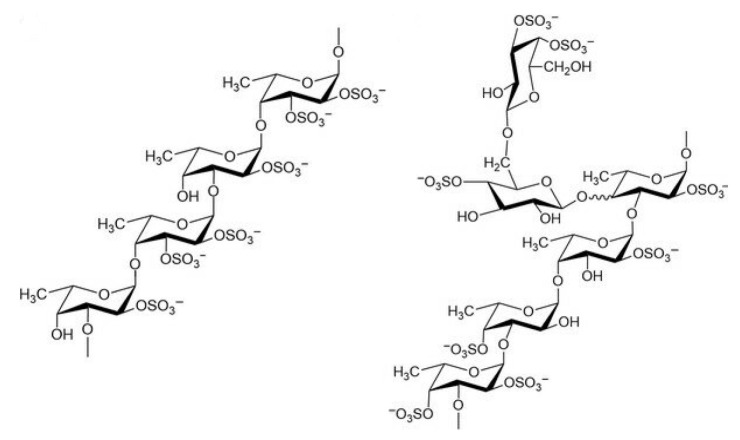
Molecular structure of fucoidan.

Moreover, chitosan is a polymer containing several functional groups that can serve as sites for chemical substitution, potentially imparting antithrombotic and antibacterial properties. The incorporation of sulfate groups into the chitosan structure improves its blood compatibility and enhances its antithrombotic activity by its complexing ability with the blood [67,68]. Chitosan sulfation can be achieved through direct modification of its repeating units at the 3-O, 6-O positions, and/or N positions using reagents such as sulfur trioxide–pyridine complex or chlorosulfonic acid in formamide. Moreover, N-sulfonated chitosan can be prepared by reacting chitosan with reagents like vinylsulfonate or propane sultone [69]. Similar to sulfated chitosan, fucoidan (Figure 5) is a natural polysaccharide characterized by a high degree of sulfation, which imparts a strong negative charge and enables interactions with proteins of the coagulation system. Fucoidan is found mainly in the cell walls of brown seaweeds (e.g., *Fucus vesiculosus*, *Laminaria japonica*) and to a lesser extent from other marine organisms.

The backbone of fucoidan is composed mainly of L-fucose units, often with considerable branching, other monosaccharides (galactose, mannose, xylose), and a substantial degree of O-sulphation (-SO_3_^−^ groups), which confer high negative charge and enable interactions with proteins of the coagulation system. From the perspective of haemostasis and thrombosis, fucoidan has attracted attention because it exhibits anticoagulant and antithrombotic effects *in vitro* and *in vivo*. Early studies showed that certain fucoidan fractions inhibited thrombin (factor IIa) and factor Xa in plasma systems, mediated in part via augmentation of natural serine protease inhibitors such as antithrombin III (AT III) and heparin cofactor II (HC II). The mechanism of fucoidan antithrombotic action is multifactorial. The literature emphasizes that the key determinants of its activity are molecular weight (M_w_), the degree and distribution of sulfate groups, and any branching of the polysaccharide chain [70,71]. Compared to heparin, fucoidan offers certain potential advantages. Primarily, as a marine polysaccharide, it may be less likely to transmit infectious agents such as viruses or prions [72]. However, despite promising *in vitro* and *in vivo* results, the antithrombotic activity of fucoidan has typically been lower than that of heparin, which poses a challenge for its development as an antithrombotic agent. Due to this, a growing number of studies are focusing on fucoidan modification (e.g., depolymerization, controlled sulfation) to obtain optimal antithrombotic properties while maintaining safety and biocompatibility [73].

**Figure 6:**
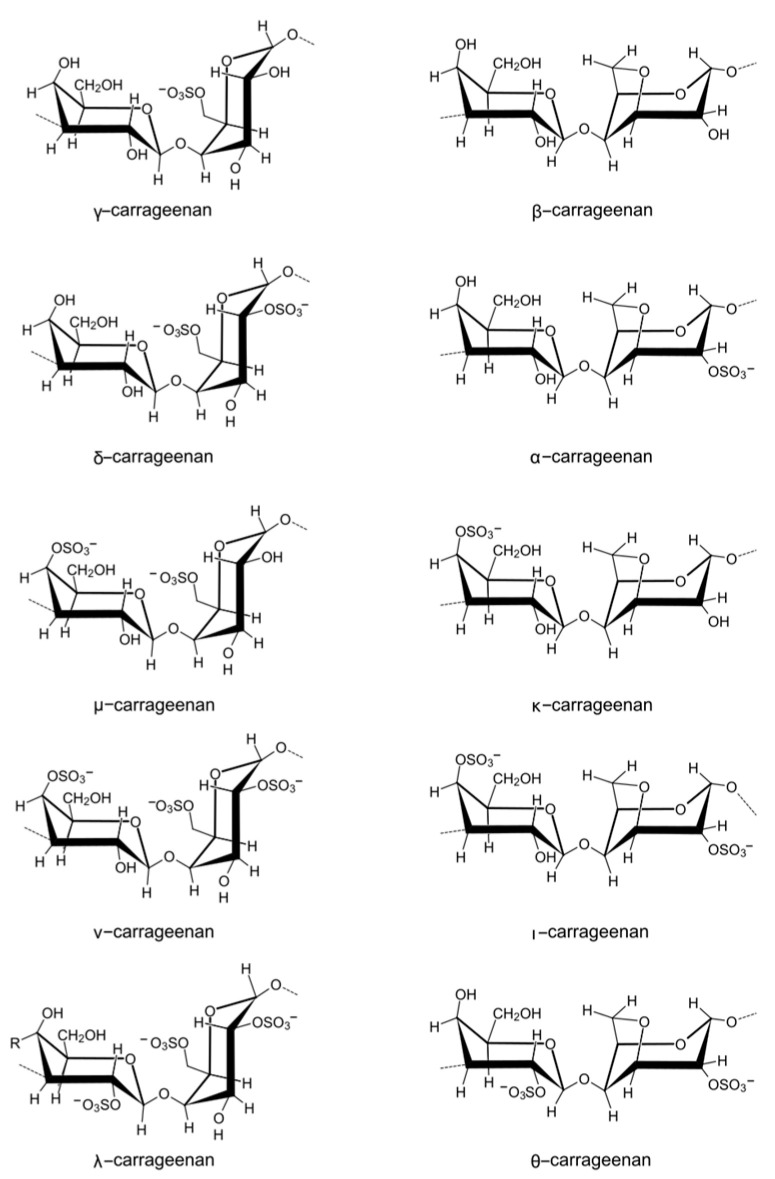
Molecular structure of carrageenans.

Similar to fucoidan, carrageenans (Figure 6) are sulfated polysaccharides derived from marine algae that exhibit notable anticoagulant potential, although their structural diversity results in distinct biological profiles. Carrageenans—linear sulfated polysaccharides isolated from red seaweeds, also known as Irish moss widely used in the food and pharmaceutical industries due to their gelling, thickening, and stabilizing properties.

Their activity is mainly attributed to the high content of sulfate groups, which confer a strongly anionic character to the polysaccharide chain and enable interactions with plasma protein [74]. Carrageenans exhibit antithrombotic activity by interacting with blood coagulation factors such as thrombin (factor IIa) and factor Xa, primarily through antithrombin III (AT-III) and heparin cofactor II. Studies have shown that λ-carrageenans have higher anticoagulant activity compared to other types of carrageenans, although their effect is significantly weaker than that of heparin [75]. Furthermore, carrageenans may affect the intrinsic coagulation pathway, as suggested by the results of aPTT and PT tests [76]. Carrageenans, due to their antithrombotic properties, represent a promising area of research in the development of new therapies and in the design of medical surfaces in contact with blood. Although their activity is weaker than that of heparin, their natural origin and the possibility of structural modifications open up new possibilities in the treatment and prevention of thrombotic diseases [77].

**Figure 7:**
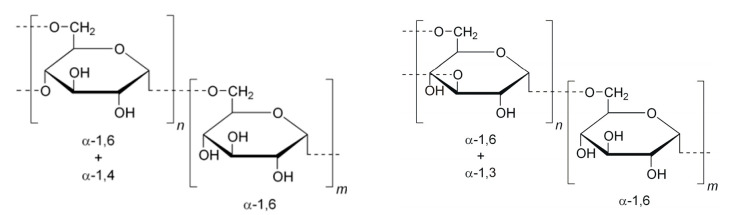
Molecular structure of dextran.

In addition to carrageenans, other natural polysaccharides such as dextran (Figure 7) have also been reported to exhibit anticoagulant and antithrombotic effects. Dextran is a neutral, high-molecular-weight polysaccharide composed mainly of α-1,6-linked D-glucose units with possible α-1,4, α-1,3, or α-1,2 bonds, produced by certain *Leuconostoc* and *Lactobacillus* bacterial strains through the enzymatic action of dextransucrase on sucrose substrates.

Commercially, dextrans of different molecular weights are widely used as plasma expanders and drug carriers due to their biocompatibility and water solubility [78,79]. Dextran also exhibits moderate anticoagulant and antithrombotic activity, though its mechanism of action differs substantially from that of heparin and carrageenans. Instead of directly inhibiting coagulation enzymes, dextran acts primarily through rheological and antiplatelet effects. It reduces blood viscosity, limits erythrocyte aggregation, and decreases platelet adhesion to vascular endothelium and biomaterial surfaces. These effects improve microcirculatory flow and prevent thrombus formation, especially in low-flow conditions. In addition, dextran slightly delays fibrin formation by interfering with fibrinogen-fibrin conversion and plasma protein interactions [33]. Although the anticoagulant potency of dextran is markedly weaker than that of heparin, its indirect and reversible action offers advantages in certain clinical settings, such as vascular surgery and microcirculatory protection, where excessive anticoagulation poses a risk [33]. Below is a table describing the comparative antithrombotic activitty of selected modifications of natural polymers (Table 2).

### 3.2. Biomimetic Synthetic Polymers

Synthetic polymers with high biocompatibility and well-controlled surface properties represent one of the most important classes of materials used to minimize coagulation activation at the blood–material interface. Unlike natural anticoagulants, these polymers do not exhibit intrinsic biochemical anticoagulant activity; instead, they act by modulating cell–material and protein–material interactions, reducing plasma protein adsorption, and disrupting the signaling cascade responsible for platelet activation. The most relevant groups include poly(ethylene glycol) (PEG), poly(ethylene oxide) (PEO), phosphocholine-containing polymers such as poly(2-methacryloyloxyethyl phosphorylcholine) (PMPC), and a broader category of amphiphilic and surface-active polymers, most commonly applied as coatings or block copolymer modifiers for blood-contacting medical devices [103].

**Figure 8:**
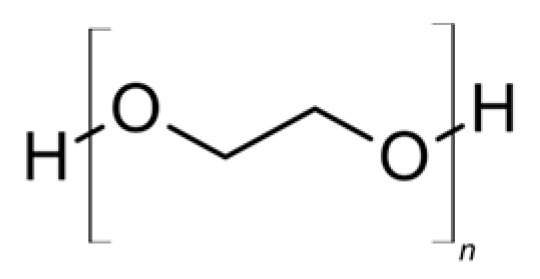
Molecular structure of poly(ethylene glycol).

Among the synthetic polymers studied, poly(ethylene glycol) (PEG) (Figure 8) has emerged as one of the most extensively explored due to its high hydrophilicity and biocompatibility, and ability to resist protein adsorption.

These characteristics make PEG a valuable component in the design of antithrombotic materials. By forming a highly hydrated, sterically repulsive surface layer, PEG effectively reduces nonspecific interactions with plasma proteins, including fibrinogen, which is a key initiator of platelet adhesion and activation. As a result, PEG-modified surfaces exhibit significantly diminished thrombogenic potential. PEG is incorporated into antithrombotic systems in several ways, including surface grafting onto vascular grafts, stents, or catheters, as well as forming hydrogels or block copolymers with other biofunctional components. PEGylation of biomaterials not only improves hemocompatibility but also enhances the circulation time of therapeutic agents by reducing opsonization and clearance. Moreover, PEG can serve as a platform for further functionalization with anticoagulant molecules, such as heparin or nitric oxide–releasing groups, synergistically enhancing antithrombotic properties. Due to its versatility, PEG remains a cornerstone polymer in the development of next-generation blood-contacting materials designed to minimize thrombosis while maintaining mechanical and biological performance [104,105,106,107].

**Figure 9:**
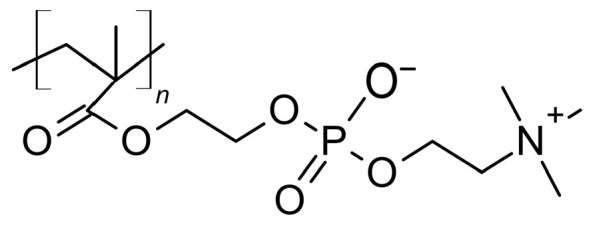
Molecular structure of poly(2-methacryloyloxyethyl phosphorylcholine).

Poly(2-methacryloyloxyethyl phosphorylcholine) (PMPC) (Figure 9) is a synthetic biomimetic polymer whose key feature is the presence of phosphorylcholine groups—identical to the main component of the outer layer of cell membranes.

This structure allows PMPC to exhibit exceptional hemocompatibility and minimal adsorption of plasma proteins, significantly limiting the initiation of the coagulation pathway on the material’s surface. The polymer forms a hydrophilic, highly hydrated layer on substrates, effectively hindering the binding of fibrinogen, albumin, and immunoglobulins, and consequently inhibiting platelet adhesion and activation. For this reason, PMPC is being widely studied as an antithrombotic coating for medical materials, including stents, vascular prostheses, catheters, and dialysis membranes. Unlike heparin coatings, its action is based on a passive “non-fouling” mechanism, requiring neither the release of active substances nor direct modulation of the coagulation cascade, which increases stability and long-term safety. The combination of resistance to protein adsorption, low thrombogenicity, and high biocompatibility makes PMPC one of the most promising polymers for creating durable, next-generation antithrombotic coatings. While highly effective, PMPC-based coatings require controlled polymerization techniques such as atom transfer radical polymerization (ATRP) or reversible addition-fragmentation chain transfer (RAFT), which can increase the cost and complexity of production [108,109]. While phosphorylcholine-based polymers such as PMPC aim to replicate the zwitterionic, non-fouling characteristics of natural cell membranes, another strategy to achieve low protein adsorption and reduced thrombogenicity relies on the intrinsic chemical inertness of fluoropolymers. Their fundamentally different mechanism of anti-fouling performance positions them as a complementary class of hemocompatible materials. Fluoropolymers have emerged as one of the most promising classes of biomaterials for blood-contacting medical devices due to their exceptional physicochemical stability and inherently low thrombogenicity. As demonstrated in multiple studies, such surfaces substantially reduce nonspecific protein adsorption, including fibrinogen and von Willebrand factor—key mediators in the early stages of thrombus formation [80]. Lower levels of fibrinogen adsorption correlate directly with decreased platelet adhesion and activation, which is considered one of the primary determinants of antithrombogenic performance *in vitro* and *in vivo* [110].

An interesting approach is the creation of entirely new polymers with functional group structures that provide antithrombotic activity. A good example of such work is the publication describing the results of research by Kałaska’s team [111], who synthesized a series of di- and triblock copolymers containing pendant sulfonic groups (polyacrylamidosulfones and polystyrene sulfonates) and PEG blocks. The resulting copolymers demonstrated potent antithrombotic activity in rodents after subcutaneous and intravenous administration. No interactions with platelets were observed, suggesting potential use in biomaterials in contact with blood for antithrombotic purposes. Further studies are necessary to fully explore its therapeutic applications.

Among fluoropolymers, PTFE and expanded PTFE (ePTFE) are the most extensively investigated. Their hydrophobic and chemically inert surfaces exhibit reduced platelet aggregation compared with conventionally used polyurethanes or polyethylene terephthalate [112]. However, unmodified PTFE still shows limitations related to restricted endothelialization and long-term biointegration. Consequently, recent research has focused on surface modifications aimed at enhancing hemocompatibility while preserving chemical stability [113,114,115]. Advances in surface engineering have led to fluoropolymer-based coatings used in vascular grafts, guidewires, stents, and catheter systems, where their ability to mitigate thrombus formation translates into improved device patency and safety [116,117]. Although fluoropolymers offer excellent chemical stability and inherently low protein adsorption, their limited elasticity and challenges related to long-term endothelialization have stimulated interest in alternative material classes. One of the most widely explored are polyurethanes (PU), which combine favorable mechanical properties with tunable surface chemistry. Polyurethanes represent one of the most versatile classes of polymers used in blood-contacting medical devices due to their favorable mechanical properties, elasticity, and ability to mimic the compliance of native vascular tissues. Nevertheless, unmodified PU surfaces readily adsorb plasma proteins such as fibrinogen and albumin, a process that can trigger platelet adhesion and subsequently promote thrombus formation. Consequently, extensive research has focused on tailoring PU chemistry and surface characteristics to improve hemocompatibility. The tunability of polyurethane materials, combined with a wide array of functional surface-modification strategies, underscores their potential as a highly adaptable and clinically relevant platform for next-generation antithrombogenic technologies, including vascular grafts, catheters, blood pumps, and stent coatings [118,119,120]. Below is presented a comparative Table 3 showing the most important synthetic polymers with antithrombogenic properties.

### 3.3. Polymers Releasing Anticoagulant Agents (Carriers for Heparin, Hirudin, Inhibitors)

Natural and synthetic biocompatible polymers are versatile biomaterials that play a crucial role in developing technologies for delivering active compounds or serving as temporary implants to treat damaged tissues or organs. Their design flexibility, achieved during the synthesis or through chemical modifications, allows for the customization of action duration to meet clinical needs. This can range from several hours, as seen with hydrogels, to weeks or months, as in the case of bioresorbable stents. Additionally, these polymers can be tailored for varying mechanical properties, from stiff to flexible [129,130]. Moreover, their unique properties enable the release of both hydrophilic and hydrophobic drugs, further enhancing the effectiveness of drug delivery systems.

**Figure 10:**
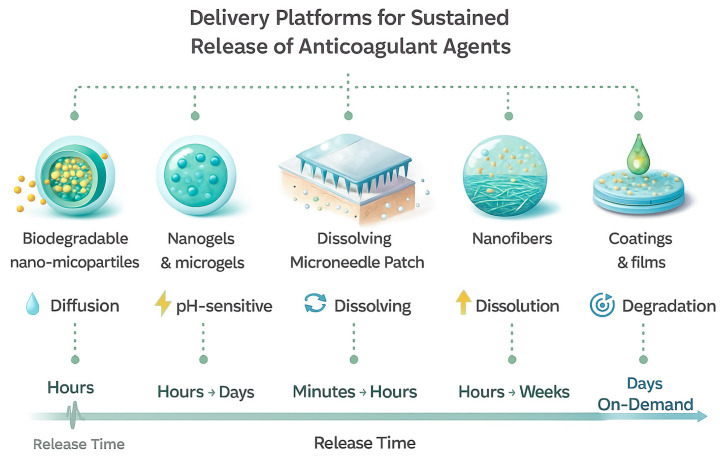
Polymeric anticoagulant delivery systems.

In recent years, a variety of systems for the release of anticoagulant agents have been investigated (Figure 10). In the thrombotic context, however, it is nanosystems that appear to exert a particularly pronounced influence on the coagulation process. Owing to advances in nanotechnology, researchers increasingly employ nanoparticles, as they are the only nanoplatforms capable of diffusing into the fibrin network of developing clots [37,131]. Many thrombotic complications develop around the material surface and within the bloodstream; therefore, the idea of controlled release of anticoagulant agents appears to be “right on target” and a highly promising direction. Such release allows the active compound to be delivered precisely to the site where the risk of thrombosis is maximal, while simultaneously reducing systemic doses and thereby lowering the risk of bleeding [132]. Thus, this concept represents a significant step forward compared with classical anticoagulant coatings: its action is not limited to the immediate material surface but also extends into the surrounding space.

The most commonly used materials in the production of systems for the release of anticoagulant agents are biodegradable polymers approved by the FDA (PLA, PLGA, and PCL) [133,134,135,136]. By using these polymers as homopolymers or as blends of copolymers, it is possible to tailor optimal mechanical properties and degradation times. Their degradation typically leads to the gradual release of the loaded anticoagulant agent, such as heparin, hirudin, fondaparinux, or Xa inhibitors. The literature describes a broad range of strategies for obtaining polymer particles from different monomers. Their morphology, stability, and suitability for specific applications depend, among other factors, on the selection and modification of the synthetic routes employed, the presence of additional stabilizing coatings (e.g., PEG), as well as the method and formulation used to obtain the delivery systems.

For nanoparticles, the resulting release profiles are usually biphasic: an initial burst release lasting up to 48 h, associated with the elution of molecules from the carrier surface, followed by a prolonged phase of slow release lasting up to 14 days. The biphasic release profile arises from hydrolytic degradation of the polymer (bulk erosion) in combination with diffusion processes [137]. Compared with PLA/PLGA, PCL provides longer stability but slower release (over months), which makes it suitable for long-term anticoagulant systems (e.g., grafts, bioresorbable scaffolds, BRS) [134]. Tyrosine-derived polycarbonates (tyrosine-based PCs) represent a third generation of bioresorbable polymers, characterized by a controlled, predictable degradation rate and the capacity to carry multiple agents (anticoagulant, antiproliferative, antibacterial) [138].

A second interesting group is polymer hydrogels, which are mainly based on natural polymers such as chitosan, alginate, gelatin and collagen, as well as synthetic PEG-based or acrylamide-based [139,140,141]. These polymers act via diffusion and swelling, regulating heparin release as a function of the degree of hydration. They typically respond to stimuli such as pH, the presence of enzymes, or even thrombin, and can release anticoagulants from their crosslinked networks in a controlled manner. Notably, this approach enables the design of “smart” systems in which release is triggered at the moment of local activation of the coagulation cascade.

In recent years, there has been growing interest in polymers that release drugs in response to specific changes in their local environment, such as elevated thrombin levels, the presence of reactive oxygen species (ROS), or acidic conditions (low pH). This approach allows anticoagulant drugs to be administered only when coagulation is actively occurring, thereby minimizing the risk of adverse effects [142]. In clinical practice, particular importance is attached to coatings made of bioresorbable polymers, which, through their degradation, enable the release of anticoagulants incorporated into the coating without the need for high systemic doses. This strategy allows the development of bifunctional systems combining antirestenotic and anticoagulant effects, which are often used together with antiproliferative agents (e.g., sirolimus) [143].

The most commonly used bioactive anticoagulant released from polymers is heparin, whose activity in its conventional form rapidly diminishes due to degradation and protein interactions. In contrast, a controlled-release formulation should allow anticoagulant activity to be maintained for longer periods at lower doses [132]. An alternative to heparin is hirudin—a direct thrombin inhibitor that acts independently of antithrombin III, and therefore retains efficacy even under conditions of high activation of the coagulation cascade [144,145]. Studies in this field have also employed fondaparinux, which selectively activates ATIII and inhibits factor Xa; in its immobilized or released form, it exhibits a favorable safety profile compared with heparin [146,147]. Furthermore, bivalirudin is being investigated as an alternative anticoagulant in cardiac surgery procedures [148]. Ongoing experiments are exploring the incorporation of newer oral inhibitors (dabigatran, apixaban) into polymers. Although optimization is still required in terms of stability and bioavailability, these systems hold promising application potential [149,150]. The objective of the present investigation was to formulate microballoons loaded with dabigatran in the polymeric core for achieving sustained release and hence improved bioavailability. Microballoons loaded with dabigatran in the polymer shell were prepared by a simple solvent evaporation method using either ethyl cellulose alone or a blend of ethyl cellulose with HPMC/PVP-K30/Eudragit S100/Methyl cellulose in a fixed ratio. The percent yield of the microballoons ranged from 38.7 to 62.4%, with the highest yield obtained in F3. The particle size was measured using optical microscopy, and the particles were observed to be spherical in shape. The particle size ranged from 28.50 ± 16.378 µm (F3) to 48.14 ± 16.748 µm (F1). The angle of repose ranged from 26.37° to 28.09°, and the Carr’s index and Hausner’s ratio were between 6.38 to 19.95 and 1.07 to 1.25, respectively. It was found that all the formulations exhibited buoyancy in the range of 61.42 to 71.71% over a period of 8 h. This suggests that the formulations were able to float for sufficient time and would be able to control the release of dabigatran for a longer duration. The *in vitro* drug release study depicted that the highest amount of drug was released from F3 (66.79%) while the lowest was released from F1 (50.27%) at the end of 8 h of study [151,152]. Despite the many advantages offered by bioresorbable polymer systems for the controlled release of anticoagulant agents, several challenging issues remain. One of the key problems is precise control of release kinetics, in particular, avoiding excessively rapid initial release, maintaining biological activity in a hydrolytic environment, and ensuring reproducibility of production on an industrial scale. Analyzing the literature reports, some of which are described in the table below (Table 4), we are still quite far from the desired optimal release model of the selected drug, ensuring the required effectiveness in a relatively long therapy period.

### 3.4. Polymers with Built-In Bioactive Function

**Figure 11:**
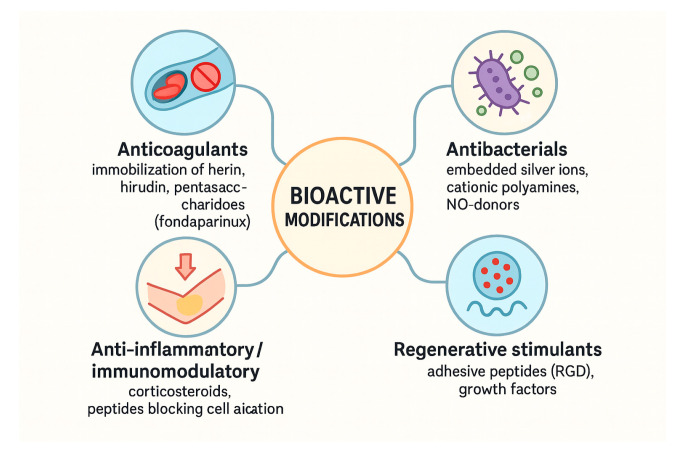
Examples of various possibilities of biomaterial modification.

Conventional polymeric biomaterials have been essential in medicine for many decades. Their widespread application primarily stems from characteristics such as biocompatibility, biodegradability, chemical stability, abrasion resistance, and favorable mechanical properties [165]. Owing to these characteristics, polymers have become the foundation for constructing a wide range of biomedical devices and artificial organs. An additional advantage is their generally good compatibility with blood and tissues, which makes them attractive materials for implants. For this reason, new strategies for polymer modification have been intensively sought for many years, aiming to improve their hemocompatibility and impart anticoagulant [166]—Figure 11.

In response to these needs, the concept of polymers with built-in bioactive function has emerged. Materials that have garnered significant interest serve a dual purpose: they provide structural support while also exhibiting biological activity. This biological activity stems from the presence of bioactive molecules or specific functional groups integrated into their structure. Unlike traditional polymers, these materials do not need additional drug carriers, as their bioactivity is inherently linked to the material itself.

One method used to modify the properties of biomaterials is physical blending, such as incorporating anticoagulant drugs or compatible dopants. While this approach can enhance the biocompatibility of vascular implants, excessive use may lead to adverse effects, including thrombocytopenia and hemorrhagic complications [167]. Despite its limitations, this method remains highly popular in clinical research because introducing bioactive compounds is relatively simple, and the resulting implants usually exhibit satisfactory properties. A key aspect of modifying biomaterials is the immobilization of biomolecules. This technique is commonly used in various fields, including molecular biology, analytical chemistry, bioprocess engineering, medical diagnostics, regenerative medicine, and tissue engineering. It allows for the precise customization of the biological and functional characteristics of biomaterial surfaces [168]. As a result, it becomes possible to design implants with markedly improved hemocompatibility and clinical safety.

Among the various modification strategies, heparin holds a significant position as one of the most extensively studied biomolecules with anticoagulant properties. Early methods for incorporating heparin involved adsorbing it onto the surface of polymers. However, this approach presented a major challenge due to the instability of the layers formed and the ease with which heparin could be washed away from the polymer. The first heparinized surfaces were described as early as the 1960s, with notable work by Gott et al., who employed heparinization in extracorporeal circulation systems [169]. In the following years, various research groups reported methods for stable and long-term anchoring of heparin to biomaterial surfaces, which markedly improved their [170,171]. The introduction of methods for covalently bonding heparin to polymers marked a significant breakthrough. This development led to a reduction in thrombotic complications associated with extracorporeal circulation systems and enhanced the safety of vascular catheters. As a result, heparin became the “gold standard” for surface modification of biomaterials designed for blood contact. Today, one of the most common techniques is the immobilization of heparin on the surfaces of various biomaterials, which effectively imparts anticoagulant properties. An overview of the most interesting works related to the discussed topic is presented in the table below (Table 5).

### 3.5. Self-Cleaning/Anti-Fouling Coatings That Reduce Platelet Activity

The previously presented clotting mechanism indicates that the properties of the blood-material contact surface are more critical for the coagulation cascade than the material itself, whether it is polymer or metal. The first response of the body to this contact is the adsorption of blood serum proteins, specifically fibrinogen, albumin, and globulin. Within seconds, this process creates a protein layer that serves as the actual interface for blood platelets and initiates the coagulation cascade [191,192]. The total protein content, along with its conformation and stability, plays a crucial role in determining whether the surface exposed to platelets is perceived as uncharged or procoagulant. Specifically, if the surface displays platelet-binding regions, such as GPIIb/IIIa for fibrinogen, and is stable and densely packed, it will influence how platelets react to the material. Therefore, the primary goal of contemporary surface modifications is not to inactivate the coagulation cascade itself, but rather to inhibit the formation of a stable procoagulant protein-platelet layer at the interface between the material and blood.

**Figure 12:**
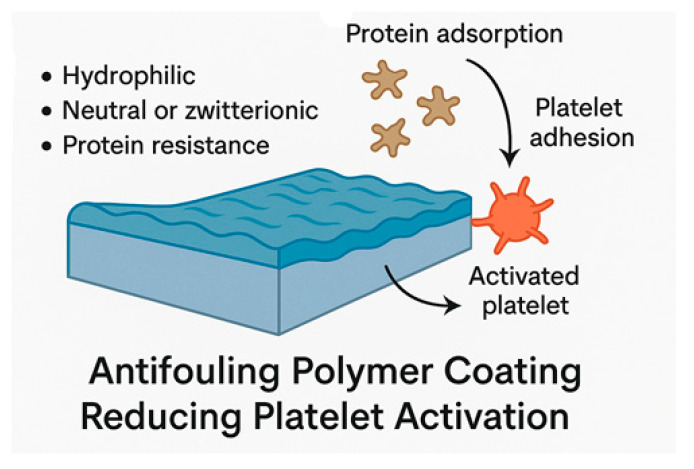
Antifouling polymer coating to limit platelet activation. The diagram shows a hydrophilic, electrically neutral, or zwitterionic polymer coating with anti-adsorption properties towards plasma proteins. Reducing protein adsorption on the implant surface reduces platelet adhesion and thus platelet activation.

As illustrated in Figure 12, classical antifouling coatings typically work by creating a highly hydrated layer at the interface. The most well-known examples of these coatings include poly(ethylene glycol) (PEG) brushes and a broad range of zwitterionic polymers, such as polysulfobetaines, carboxybetaines, and phosphorylcholine-containing copolymers [193,194,195]. In the hydrated state, PEG chains form a dense, mobile network that binds large amounts of water; bringing a globular protein close to such a layer is associated with an unfavourable loss of entropy and strong steric repulsion, which translates into a several-fold to more than 90% reduction in protein adsorption compared with unmodified surfaces. A similar effect, although based on a different mechanism, is observed for zwitterionic brushes of poly(sulfobetaine methacrylate) (poly SBMA) and related systems: strongly hydrated ion pairs (a cationic and anionic group within the same monomer unit) form so-called “intermediate water”, which very effectively screens electrostatic and hydrophobic interactions between proteins and the surface [196]. Numerous studies have demonstrated that sufficiently dense zwitterionic brushes on silicone, stainless steel, or porous membranes can almost eliminate fibrinogen adsorption and markedly reduce the number of adherent platelets, both in simple static assays and under *ex vivo* flow conditions.

An excellent example of a clinically oriented antifouling coating is the use of phosphorylcholine-based polymers that mimic the outer layer of the cell membrane. The PMBT copolymer (poly(2-methacryloyloxyethyl phosphorylcholine-co-n-butyl methacrylate-co-3-(trimethoxysilyl)propyl methacrylate)), tethered to polymethylpentene membranes used in ECMO oxygenators, forms a stable, highly hydrophilic layer that is resistant to washing with ethanol and detergents [197,198]. Whole-blood experiments have shown a significant reduction in the number of adherent platelets and in thrombus thickness on PMBT-coated membranes compared with uncoated ones, while preserving gas-exchange performance [199]. A similar effect has been observed for nickel–titanium stents coated with a hydrophilic HPC-II (2-hydroxypropyl cellulose) layer: the proportion of CD61-positive platelets on NiTi surfaces decreased from approximately 40–50% for the uncoated material to about 1% for the HPC-II coating, as confirmed by both flow cytometry and scanning electron microscopy [200]. These findings clearly illustrate that modifying surface wettability and the “chemistry of water” at the blood–material interface can substantially reduce platelet activation without the need for permanently immobilised anticoagulants.

In current clinical practice, hydrophilic and zwitterionic solutions are predominant because they can be easily integrated with existing technologies and demonstrate greater chemical and mechanical stability. Reviews of the hemocompatibility of zwitterionic interfaces highlight that increasing the brush density to several chains per square nanometer leads to almost complete suppression of fibrinogen deposition, a minimal presence of adherent platelets, and very low activation of the complement system. Additionally, “selective antifouling” coatings are emerging that effectively repel plasma proteins and platelets while preserving—or even promoting—adhesion of endothelial cells. These systems, which may be based on zwitterionic brushes functionalized with adhesive motifs, represent a promising approach for developing vascular grafts and stents that support rapid endothelialization while simultaneously protecting against early thrombosis [201,202,203,204].

The common thread among the approaches described is the goal of minimizing the development of a stable, prothrombotic protein–platelet layer on the surface of implants. Antifouling coatings achieve this by creating a persistent hydration layer and utilizing steric or electrostatic repulsion to prevent protein adhesion. In contrast, self-cleaning coatings minimize the actual contact area and facilitate the easy removal of contaminants. While both strategies have their limitations—such as PEG degradation, the susceptibility of superhydrophobic surfaces to fouling and shear, and the stringent requirements for high-density zwitterionic brushes—they provide a strong foundation for developing hybrid systems [191,205]. In the realm of polymer-based antithrombotic materials, self-cleaning and antifouling coatings play a vital role as the first line of defense. By significantly reducing the likelihood of clot formation, these coatings not only enhance safety but also minimize the strain on the bioactive layer and the drug-release system integrated into the material. Investing in these innovative coatings can lead to more effective treatments and improved patient outcomes [206,207]. Examples of such actions are presented in Table 6.

## 4. Strategies for Chemical Surface Design and Modification

**Figure 13:**
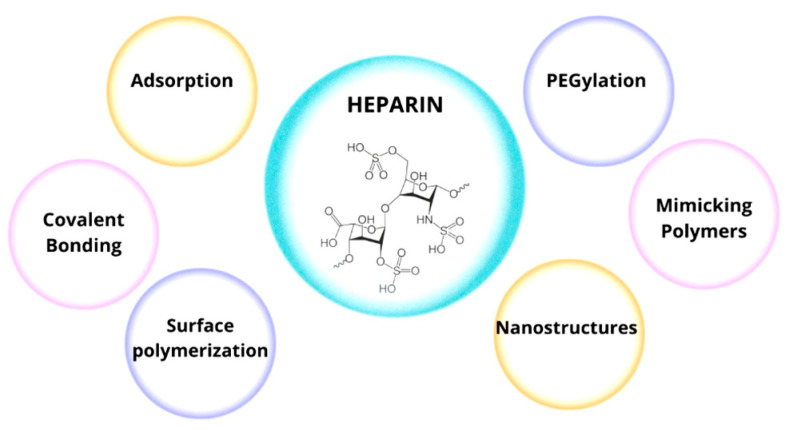
The most common direction of chemical modification of heparin and its mimetics.

The advancement of synthetic materials intended for contact with blood or tissue presents a crucial challenge: achieving exceptional biocompatibility while offering a diverse array of biological functionalities. When a biomaterial interacts with blood or tissue, it initiates a cascade of biological reactions at its surface. These reactions can lead to problematic protein adsorption, platelet activation, and the coagulation cascade, as well as inflammatory responses and the risk of immunological rejection. Furthermore, there are concerns about potential long-term toxic or mutagenic effects. Overcoming these hurdles is essential for the development of safe and effective biomaterials that can revolutionize medical applications [178,217,218]. Many of these adverse events originate from suboptimal physicochemical properties of the material or from an inadequately designed biointerface; therefore, numerous surface-modification strategies have been proposed and tested in recent decades. The most common approaches include chemical immobilization of bioactive agents, plasma surface treatment, application of coatings, grafting of functional (bio)polymers, creation of nanostructures, and various biologically inspired methods (Figure 13).

### 4.1. Heparin Functionalization (*Adsorption vs. Covalent* Bonding)

**Figure 14:**
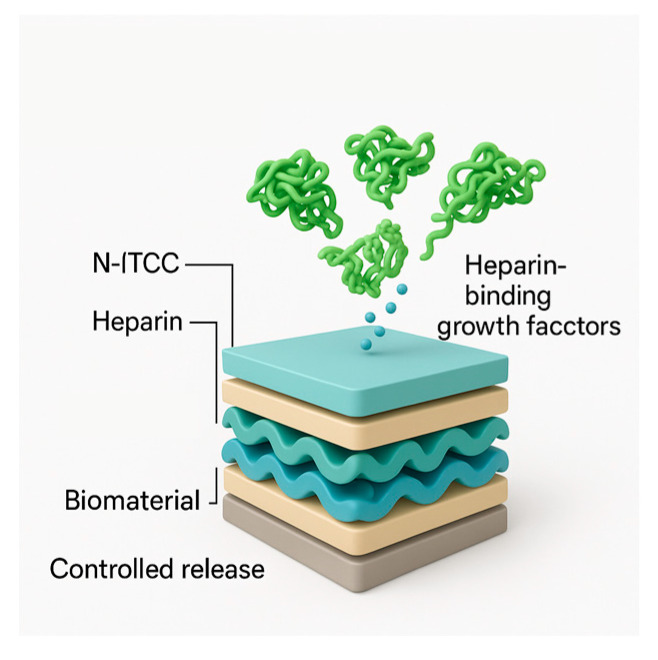
Heparin-based LBL systems.

Heparin and its mimetics are gaining increasing attention as functional components of biomaterial coatings due to their anticoagulant properties, ability to bind growth factors, and relatively good biocompatibility [178,215,216,218]. Heparin immobilization on biomaterial surfaces can generally be achieved through two main strategies: adsorptive methods (including layer-by-layer—LbL) and covalent bonding. Adsorptive approaches utilize electrostatic and hydrophobic interactions, often mediated by an initial protein adsorption layer. The advantages of these methods include simplicity and the preservation of the native conformation of the heparin chain, which helps maintain biological activity and allows for the retention of growth factors. The LbL method (Figure 14), in particular, enables the modular design of thin films with controlled sequence and thickness, making it easier to incorporate protein reservoirs and regulate signal release [219]. However, the main limitation is poor resistance to wash-out under flow conditions and the risk of conformational changes triggered by strong electrostatic interactions—potentially leading to loss of accessibility of critical domains (e.g., the ATIII pentasaccharide). In practice, hybrid solutions (adsorption and pointwise covalent anchoring), protein preadsorption, or porous carriers that increase the number of physical interactions are employed; however, all of these require application-specific validation [132,219,220]. Research clearly highlights the remarkable versatility of layer-by-layer (LbL) heparin systems.

In their 2019 study, Zhang et al. pioneered the development of polyelectrolyte vascular coatings through LbL deposition of heparin and chitosan onto polyurethane-decellularized scaffolds (PU/DCS). These innovative coatings not only improved biocompatibility but also significantly extended clotting times, reduced hemolysis, and lowered platelet adhesion—all while effectively targeting endothelial progenitor cells. Most notably, the heparin-based LbL coatings ensured vessel patency for up to five months post-implantation, a stark contrast to the rapid occlusion seen in non-functionalized PU/DCS scaffolds, which failed within days. These compelling findings strongly advocate for the use of LbL heparin coatings as superior, long-lasting interfaces for vascular applications [221]. Heparin’s potential to modulate the healing environment was also confirmed by Nawaz et al., who functionalized commercial alginate dressings with heparin. Heparin’s natural ability to swell and form hydrated gels enhances the capacity of dressings for controlled water release and exudate absorption. Importantly, this modification also boosts the angiogenic potential of the substrate and exhibits antibacterial activity against *E. coli* and *S. aureus*—two key pathogens that complicate the healing of chronic wounds. These findings suggest that incorporating heparin into wound dressings may not only support the early stages of regeneration but also help modulate the wound microenvironment to reduce the risk of infection and accelerate healing [178].

Heparin-based coatings present exciting opportunities in biomaterials designed as protein reservoirs. A notable study by Urbaniak et al. introduced stable, bioactive layer-by-layer (LbL) films made from N-[(2-hydroxy-3-trimethylammonium)propyl] chitosan chloride (HTCC), heparin, and tannic acid (TA). TA acts as a crosslinker, enhancing structural integrity and allowing for precise control over protein release. These multilayered structures effectively regulate the release of heparin-binding growth factors like VEGF, CXCL12, and TGF-β1 while preserving their biological activity. The research demonstrated long-term release profiles of up to four weeks, making them highly beneficial for tissue regeneration. Additionally, their lack of cytotoxicity and ability to stimulate T-cell chemotaxis indicate that heparin-based LbL systems can effectively support the development of advanced immunomodulatory biomaterials [222]. A highly advanced LbL medical application was presented by Lee et al., who engineered LbL coatings for angioplasty balloons incorporating heparin and paclitaxel-loaded albumin nanoparticles. Heparin provided the negative charge needed for electrostatic assembly and improved hemocompatibility during PCI procedures. *In vivo*, the coating remained stable during balloon inflation and released paclitaxel effectively at the targeted vascular site, significantly reducing restenosis risk. This work illustrates heparin’s multifunctional role—not only anticoagulant but also structural and electrostatic—in interventional drug-delivery systems [223].

Covalent immobilization ensures great durability in dynamic environments. Heparin can be attached either directly or through linkers using carboxyl activation and amide-bond formation with EDC/NHS. The choice between end-point attachment (EPA) and multipoint attachment (MPA) is essential: EPA minimizes chain stiffening while preserving natural conformation, whereas MPA enhances mechanical stability at the cost of some chain mobility. Incorporating flexible spacers (linkers) can help balance this trade-off for optimal performance [224,225,226,227]. One of the pioneering examples of covalent immobilization is the study by Wissink et al., which successfully immobilized heparin on a collagen substrate using EDC/NHS. This method resulted in improved blood compatibility and reduced platelet activation. The research showed that increasing the molar ratio of EDC to heparin carboxyl groups enhances immobilization. However, this can also cause chain stiffening and partial loss of access to active domains [228].

In the following years, heparin immobilization was extended to more complex polymer matrices and layered constructions. Elahi and colleagues performed immobilization of EDC/NHS-activated heparin on polyelectrolyte-modified fibroin fabric using the LbL method. They demonstrated that applying PAH/PAA layers enables uniform distribution of heparin across the rough, hierarchically structured surface, improving its hydrophilicity and markedly reducing platelet adhesion. These studies highlighted that immobilizing heparin on silk-fibroin-based platforms offers a realistic pathway toward developing small-diameter vascular grafts with enhanced hemocompatibility [224,225,226,227,229,230,231,232,233].

A significant advancement in heparin functionalization involved differentiating between two covalent strategies: end-point attachment (EPA) and multipoint attachment (MPA). Bao et al. compared these two approaches, along with electrostatic immobilization, within decellularized liver scaffolds. They found that while EPA is less stable than MPA, it preserves the highest bioactivity of heparin due to minimal chain stiffening. In contrast, MPA offers greater mechanical robustness and flow stability, making it ideal for systems subjected to intense hemodynamic loads. Despite their differences, both strategies resulted in very low platelet activation and demonstrated high compatibility with endothelial cells, showcasing the versatility of covalent approaches [230]. Applications involving the functionalization of synthetic scaffolds, such as poly(ε-caprolactone) (PCL), are equally important. In a study by Xu et al. two methods were described for introducing heparin onto electrospun PCL scaffolds. These methods utilize hexamethylene diamines or L-lysine as surface-eroding reagents. This process generates amine groups that facilitate the coupling of heparin [224]. Their results revealed that subtle differences in the chemistry of the covalent linkage can affect the amount of immobilized heparin, clotting time, and endothelial cell proliferation—factors crucial for designing vascular scaffolds [224,231,232].

In recent years, research has increasingly focused on three-dimensional constructs based on hyaluronan (HA), poly(ethylene glycol) (PEG), or their mixtures, in which heparin functions both as a bioactive module and as a regulator of growth-factor release. A representative example is the work of Palumbo et al., who demonstrated that incorporating heparin as a cytokine-binding element within an HA hydrogel significantly enhances signal-molecule retention and modulation of regenerative responses. These studies open a new chapter in the use of heparin as a stable, chemically anchored carrier for therapeutic biological cues [233].

Szczubiałka’s team presented another method that could potentially control the properties of heparin derivatives in the future, thereby mitigating the negative effects of their use [82]. Unfractionated heparin (UFH) or enoxaparin (medium molecular weight heparin) was modified by covalent binding to a specially selected photoswitch (PS) exhibiting quantitative trans-cis and cis-trans photoisomerization. Appropriate irradiation altered the geometry of the resulting conjugates, potentially altering their anticoagulant and cytotoxic properties. The data obtained indicate that it is possible to achieve photocontrol of some biological activities of heparin, even with a relatively small degree of PS substitution, while simultaneously reducing its antithrombotic activity, which could open up new applications for this drug.

Both adsorption and covalent immobilization offer unique advantages and limitations. Adsorption—especially LbL—preserves conformation and bioactivity but often lacks stability under flow, necessitating additional reinforcement strategies. Covalent immobilization ensures durability and predictable performance but requires precise control of coupling density to prevent conformational distortion and loss of biological activity. Future advances will likely exploit hybrid architectures—combining covalent anchors with LbL reservoirs or PEG spacers—to achieve selective growth-factor retention, tunable release, flow resistance, and minimal distortion of heparin’s bioactive motifs. Such systems hold promise for developing next-generation biomaterials with integrated antithrombotic, pro-angiogenic, and immunomodulatory functionalities.

### 4.2. Functionalization by Surface Polymerization: Grafting from and Grafting to

**Figure 15:**
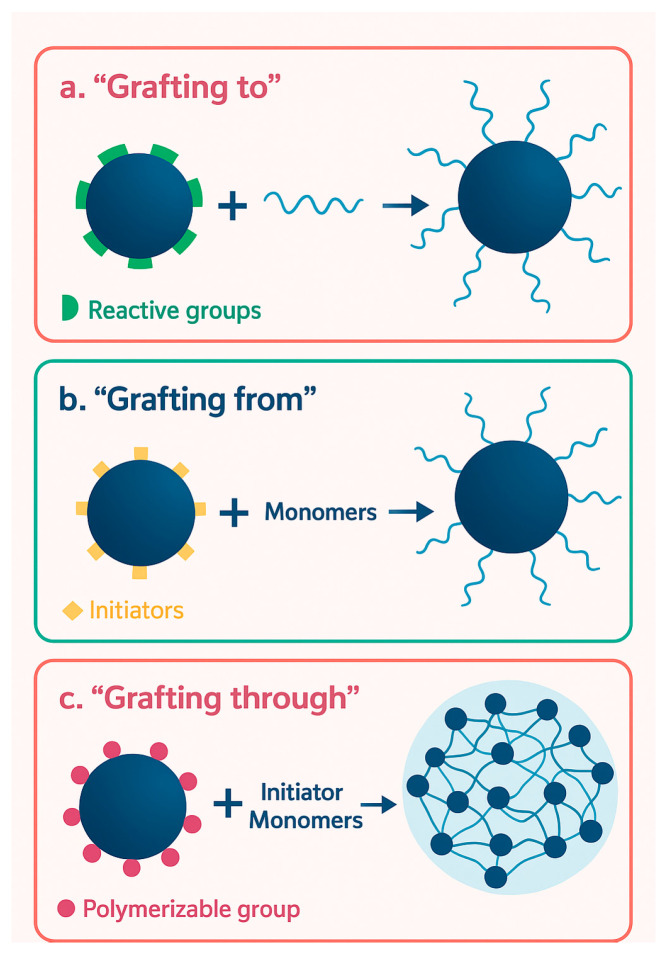
Principles of polymer immobilisation on the surface by grafting.

Modern strategies for surface functionalization of biomaterials with heparin rely on two complementary approaches—grafting to and grafting from—which enable controlled, covalent anchoring of heparin or heparin-like structures onto polymeric, metallic, or hydrogel substrates (Figure 15). Both methods allow precise tailoring of anticoagulant, proangiogenic, anti-inflammatory, and microenvironment-regulating properties; however, they differ substantially in mechanism and in the degree of control over the architecture of the surface layer [234,235]. The grafting approach is based on chemically attaching pre-activated heparin molecules to a surface presenting reactive groups, most commonly carboxyl, amine, or hydroxyl functionalities.

A variety of coupling techniques can be employed to form stable and durable covalent bonds. These techniques include EDC/NHS-mediated activation, Schiff-base formation, click reactions, aminolysis, and multipoint crosslinking using aldehydes or epoxies. One of the main advantages of this approach is that it preserves the native structure of heparin, allowing it to maintain its full biological activity. This includes strong anticoagulant properties, a high affinity for growth factors (such as FGF-2, VEGF, and BMP-2), inhibition of platelet adhesion and activation, and the regulation of extracellular matrix (ECM)-mediated signaling [234,235]. Heparin layers produced via grafting-to exhibit high stability under both static and flow conditions, making this strategy highly suitable for vascular coatings, implantable materials, and long-term release systems for bioactive molecules. An illustrative example is the work of Li et al., in which heparin was activated with EDC/NHS and subsequently conjugated to a chitosan-based hydrogel network via a click reaction. The resulting material displayed increased anionic character and strong affinity for VEGF and FGF-2, enabling their long-term stabilization and controlled release. These hydrogels demonstrated substantial proangiogenic and regenerative potential, particularly relevant to wound healing and tissue engineering applications [226]. Additional examples include the studies by Xu et al., who employed grafting-to of heparin onto aminolyzed PCL fibers, enabling efficient covalent anchoring of heparin chains to electrospun PCL matrices [231]; Elahi et al., who used a hybrid LbL + grafting to (EDC/NHS) approach to modify silk-fibroin fabrics [227]; and Palumbo et al., who introduced the concept of heparin-crosslinked HA sponges, in which heparin serves as a chemical crosslinking element forming three-dimensional porous hyaluronan scaffolds. Together, these examples highlight the versatility and reproducibility of grafting-to as a universal method for heparin immobilization, particularly valuable when preservation of full biological activity is required [233].

The grafting-from method is a highly sophisticated approach that utilizes heparin or another glycosaminoglycan (GAG) as a macroinitiator or macro–chain-transfer agent (macro-CTA). This innovation allows for the controlled polymerization to initiate directly from its structure, offering unparalleled advantages. Unlike the grafting-to technique, which faces limitations due to steric hindrance and the diffusion of macromolecules toward the surface, grafting-from allows for the development of exceptionally high-density, long polymer brushes with uniform surface coverage. This breakthrough technique employs controlled polymerization methods such as surface-initiated Atom Transfer Radical Polymerization (ATRP), Reversible Addition–Fragmentation Chain Transfer Polymerization (RAFT), UV initiation, or redox initiation. Each of these methods ensures precise control over brush architecture, making grafting-from a superior choice for advanced applications [234,235]. In the context of heparin-based materials, grafting-from enables creation of hybrid, multifunctional interfaces that are chemically stable, degradation-resistant, and retain all intrinsic properties of heparin while acquiring new functionalities introduced by synthetic polymer chains—such as anti-adhesive behavior, environmental responsiveness, or controlled growth-factor presentation. Paluck et al. presented an elegant example of a heparin-mimetic block copolymer synthesized via RAFT polymerization in which sulfonated segments showed a strong ability to stabilize FGF-2 and enhance its biological activity. Although synthesizing polysaccharide-based macro-CTAs is complex, the authors demonstrated that such materials can achieve heparin-like functionality with significantly greater structural predictability [236]. A more direct form of true heparin grafting-from was demonstrated by Pilipenko et al., who converted GAGs—including heparin—into macro-CTAs capable of controlled RAFT polymerization. This enabled “growing” poly(N-isopropylacrylamide) (PNIPAM) chains of defined length and narrow molecular distribution directly from the heparin molecule, yielding hybrids with temperature- and pH-responsive behavior. These materials combined the bioactivity of GAGs with the tunability of smart synthetic polymers, making them promising candidates for advanced drug-delivery systems and dynamic biomaterials responsive to microenvironmental cues [237].

Grafting to and grafting from are two complementary strategies used for functionalizing heparin, allowing for the precise engineering of biomaterials with controlled bioactivity and stability. Grafting is a straightforward method that preserves the native properties of heparin and is compatible with a wide range of substrates, making it a preferred choice for traditional heparin coatings. In contrast, grafting from enables the creation of highly organized polymer brushes, providing fine architectural control and access to functionalities beyond what heparin alone can offer. This approach opens up new possibilities for designing advanced interfaces in both heparin-based and heparin-mimetic biomaterials. A summary comparing the advantages and disadvantages of methods to mobilize heparin on surfaces is presented in the table below (Table 7).

### 4.3. Heparin Functionalization with PEG

In recent years, the functionalization of heparin with poly(ethylene oxide)—PEG, has proven to be one of the most powerful strategies for significantly enhancing the stability, biological activity, and biocompatibility of heparin-based systems. PEG boasts remarkable properties such as excellent water solubility, high conformational flexibility, the formation of strongly hydrated layers, and low immunogenicity, making it an ideal candidate as both a spatial buffer that effectively separates heparin from the biomaterial surface and as a “stealth” module that skillfully shields the system from immune responses. By maintaining chemical neutrality and adopting a random-coil conformation in solution, PEG generates a large hydrodynamic volume that creates a protective, water-rich shield around the molecule. This hydrated and sterically resistant PEG layer effectively prevents undesirable outcomes like protein adsorption, opsonization, and enzymatic degradation. Consequently, this translates into extended circulation times for conjugates, enhanced accessibility to bioactive heparin domains, and a more predictable pharmacokinetic profile, ultimately improving the effectiveness of heparin-based therapies [238,239]. In heparin-based applications, polyethylene glycol (PEG) plays a crucial role by stabilizing the heparin structure and protecting it from oxidative and enzymatic degradation. It also helps minimize undesired interactions with serum proteins and reduces burst release from biomaterial surfaces. Selecting an appropriate PEG chain length and architecture—such as linear, branched, or star-shaped—enables precise control over heparin orientation, accessibility to antithrombin III, and the inhibition of platelet activation. As a result, PEGylated heparin systems behave like a “dynamic polymer brush,” in which heparin is both shielded and functionally optimized. These advanced materials show strong potential for use in vascular engineering, surface modification technologies, and controlled drug delivery systems. Recent advances in PEG–heparin conjugation have highlighted several key factors that influence system performance. The surface packing density of heparin is critical for maintaining accessibility to its active domains, while the PEG chain length and molecular weight also play major roles. Furthermore, the conjugation strategy—whether direct coupling or linker-mediated—as well as PEG architecture, affects overall functionality. High packing density can enhance resistance to elution, but excessive crowding may limit access to antithrombin III (ATIII)-binding sequences. PEG chains in the 2–10 kDa range generally provide an optimal balance between mobility and stability. Additionally, star-shaped or multi-arm PEG constructs can form hydrogel networks that promote uniform heparin distribution, thereby extending its anticoagulant and pro-angiogenic effects. Together, these innovations hold strong promise for significantly improving therapeutic outcomes [240,241,242,243]. Zhu et al. demonstrated one of the most promising examples of PEG and heparin integration in the design of compliant vascular grafts. Their electrospun poly(ester-urethane)urea (PEUU) grafts were functionalized via covalent PEG–heparin conjugation, resulting in materials that preserved the mechanical properties of native vessels while exhibiting excellent resistance to platelet activation and strongly promoting endothelial cell adhesion and proliferation. *In vivo*, the PEUU@PEG-Hep grafts rapidly endothelialized and remained patent, marking an important step toward clinical translation [243].

Significant advances have been made in PEG–heparin hydrogels, particularly as long-lasting antithrombotic coatings. Research conducted by Zhang et al. showed that poly(ethylene glycol) diacrylate (PEGDA) and poly(ethylene glycol) methacrylate (PEGMA) hydrogels, when appropriately adjusted for crosslinking density, were able to continuously release heparin for up to one week. These hydrogels effectively inhibited platelet adhesion and prolonged plasma recalcification time. Such systems hold great promise for use as coatings in Extracorporeal Membrane Oxygenation (ECMO), where thrombogenicity poses a significant clinical challenge [234]. PEG–heparin systems also play an emerging role in the engineering of biodegradable materials. Hong et al. showed that the immobilization of 6-arm PEG–heparin on magnesium alloys not only significantly improved corrosion resistance but also increased hydrophilicity, reduced platelet activation, and enhanced endothelial cell proliferation—paving the way for Mg-based vascular scaffolds with controlled degradation [235].

Emerging evidence firmly establishes PEG functionalization as a leading-edge and highly promising strategy in the evolution of heparin-based biomaterials. As we look to the future, we anticipate two pivotal directions: (1) the innovation of stimuli-responsive PEG–heparin systems (including redox-, pH-, or enzyme-responsive variants) tailored for precise activation at pathological sites; and (2) the engineering of hybrid coatings and hydrogels that incorporate PEG, heparin, and other polymers or peptides into sophisticated structures with programmable bioactivity. These advanced integrated systems hold the potential to revolutionize clinically relevant biomaterial solutions, providing essential functions that include not only anticoagulation, but pro-angiogenesis, immunomodulation, and targeted therapy too. Table 8 below summarizes selected works on the functionalization of heparin through PEGylation.

### 4.4. Heparin-Mimicking Polymers

**Figure 16:**
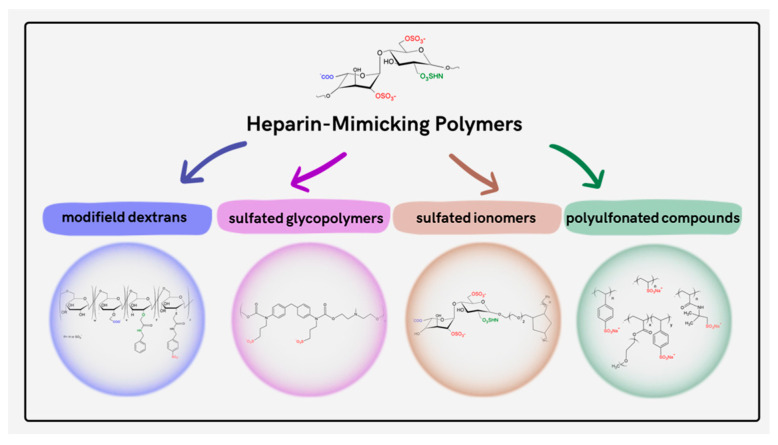
Methods for forming heparin mimetics.

Engineering of heparin-mimetic polymers has, in recent years, become one of the fastest-growing areas in the design of bioactive materials, enabling the recreation of key heparin functions while eliminating its major limitations, such as structural heterogeneity, dose variability, and the risk of adverse effects. Strategies for synthesizing heparin mimetics include the production of structurally defined oligosaccharides and their derivatives, chemical sulfonation of natural polysaccharides—such as chitosan and hyaluronic acid—as well as the design of fully synthetic sulfonated polymers or glycopolymers with precisely controlled distribution of functional groups (Figure 16). These systems can be manufactured with extremely narrow molecular-weight distributions, controlled degrees of sulfonation, and architectures tailored to specific biological functions, enabling modulation of affinity for heparin-binding proteins, resistance to degradation, and stability under physiological conditions [236].

A key advantage of synthetic heparin mimetics is their resistance to enzymatic degradation by heparinases, which significantly prolongs their activity *in vivo*. Depending on the therapeutic application, this can be highly beneficial, although it may also require the incorporation of biodegradable polymer segments to ensure proper clearance. Moreover, the ability to introduce reactive functional groups—absent in natural heparin—opens the door to designing conjugates, hybrid systems, and materials that respond to environmental stimuli. For these reasons, numerous research groups are working to develop synthetic mimetics as more reproducible, purer, and safer alternatives to animal-derived heparin, often achieving biological activity equal to or even greater than that of the natural molecule [247,248]. The most advanced class of these materials comprises polymer “brushes” based on poly(sulfopropyl methacrylate) (PSPMA) and poly(styrenesulfonate) (PSSS). Synthesized via controlled polymerization techniques, predominantly SI-ATRP, they form densely packed anionic layers capable of stabilizing and retaining growth factors such as BMP-2, FGF-2, and VEGF [234].

Marchena et al. demonstrated that surfaces coated with PSPMA brushes recreate a glycosaminoglycan-like microenvironment, effectively presenting Bone morphogenetic proteins (BMPs) and modulating ECM remodeling, making them a promising platform for tissue engineering [239]. Comparable findings were reported by Bray (2018), where sulfonated methacrylate copolymers protected FGF from degradation and enhanced its biological activity—an effect governed not only by charge density but also by polymer architecture and the spatial distribution of sulfonate groups [249].

An interesting subgroup of mimetics consists of block copolymers synthesized via RAFT polymerization, such as the styrenesulfonate–PEG-methacrylate block systems described by Paluck et al. These materials exhibit heparin-like properties in FGF-2 stabilization, support endothelial cell migration, and maintain receptor-mediated signaling, while remaining chemically uniform and free from the batch-to-batch variability characteristic of natural heparin. Synthetic heparinomimetics increasingly include zwitterionic copolymers based on sulfobetaines. Owing to the synergy between electrostatic neutrality and high anionicity following sulfonation, these materials combine resistance to nonspecific protein adsorption with selective growth factor binding [250]. Heide et al. summarize the development of synthetic heparin mimetics and their impact on the extracellular matrix. They describe how sulfated polymers interact with ECM proteins and regulate growth factor binding. Owing to their controlled structure, these mimetics can modulate matrix remodeling, angiogenesis, and cellular signaling. The authors emphasize that synthetic heparin analogues offer greater reproducibility and precision than natural GAGs, making them valuable tools in tissue engineering [251].

As shown by Wu et al., sulfated poly(sulfobetaine methacrylate) (SBMA) hydrogels create microenvironments favorable for angiogenesis and skin regeneration [252], while enhanced PSBMA-based versions (Xiao et al.) provide long-term, stable FGF-2 release, confirming their potential as bioactive therapeutic platforms [253].

Synthetic glycopolymers designed to replicate key sulfation motifs of heparin and heparan sulfate are gaining importance. With precise control over chain length, sulfation pattern, and polymer topology, these glycopolymers can mimic highly specific GAG–protein interactions [65]. Examples include polymers described by Abdulsalam et al., capable of selectively binding the SARS-CoV-2 spike protein in a sulfation-pattern-dependent manner—a finding of fundamental relevance for antiviral HS mimetics [254]. Similarly, advanced glycopolymers presented by Kardeby et al. (2019) [255] interact with platelet receptors PEAR1 and GPIbα, showing that appropriate local densification of sulfonate groups can modulate hemostatic functions, enabling the design of selective coagulation regulators. The work of Lok and colleagues further demonstrates that glycopolymers with tailored sulfation patterns can act as heparanase inhibitors, protecting β-cells from ECM degradation and inflammation [255].

A large group of mimetics is also derived from naturally occurring polysaccharides subjected to sulfonation, especially HA and chitosan. Sulfonation enables charge densities approaching that of heparin while maintaining high biocompatibility and tunable degradability. Sulfonated chitosan derivatives, as shown by Kocabay et al., interact strongly with heparin-binding proteins and exhibit antimetastatic and anticoagulant potential [256]. Sulfonated HA hydrogels (Revuelta et al. and Feng et al.) improve growth-factor retention, stabilize TGF-β, and support human mesenchymal stem cells (hMSC) chondrogenesis while limiting hypertrophy—a common problem in classical differentiation protocols [257,258].

A separate and increasingly influential category involves fully synthetic heparin-like polymers lacking any saccharide backbone, based instead on sulfonated polypeptides, polyesters, or polyglycerols. These systems combine high anionicity with full architectural control, enabling modulation of interactions with ATIII, growth factors, and immune receptors. Examples include the sulfonated polypeptides developed by Lu et al. (2021), which show predictable anticoagulant properties dependent on sulfonation degree, with structural uniformity minimizing adverse effects such as Heparin-Induced Thrombocytopenia (HIT) [259]. Even more advanced multifunctional sulfonated polyesters—such as cinnamaldehyde (CA)-based sulfonated all-polyester prodrug (RCSAP) described by Liu et al.—integrate heparin-mimetic activity with ROS-responsiveness and antibacterial properties, making them a promising translational platform for infection control and inflammation regulation [260].

One of the most recent studies, conducted by Liu et al., developed a heparin-like coating based on dendritic and linear sulfonated polyglycerol, forming a durable, highly anionic, and hemocompatible layer. The coating reduces platelet activation, protein adsorption, and inflammatory responses, and remains functional for over 30 days, making it a promising and stable replacement for natural GAGs in blood-contacting devices [261].

Advances in the design of synthetic heparin mimetics indicate a clear shift from heterogeneous natural polysaccharides toward materials with precise, molecularly controlled architectures capable of faithfully reproducing key heparin functions while eliminating its inherent limitations. Synthetic polymers—including PSPMA/PSSS polymer brushes, sulfonated glycopolymers, zwitterionic hydrogel networks, sulfonated natural polysaccharides, and the new generation of sulfonated polypeptides, polyesters, and polyglycerols—enable independent tuning of charge density, degree and topology of sulfation, chain length, and degradability. Collectively, research shows that these materials can replicate the most critical biological functions of heparin, including (1) stabilization of growth factors (FGF-2, VEGF, BMP-2), (2) modulation of the ECM microenvironment and angiogenesis, (3) protection against HS-degrading enzymes, (4) anticoagulant activity mediated by ATIII interactions, and (5) selective binding of heparin-binding proteins at levels comparable to or even exceeding natural heparin.

At the same time, synthetic mimetics exhibit higher predictability, chemical stability, enzymatic resistance, and safety, facilitating their translation into next-generation biomaterials such as tissue-engineering scaffolds, controlled-release systems, implant coatings, and therapeutic heparanase inhibitors. Particularly promising are structures capable of combining multiple functions synergistically—for example, ROS-responsive heparin mimetics or copolymers integrating zwitterionic behavior with high anionicity.

In light of current evidence, synthetic heparin mimetics emerge not as substitutes but as platforms that functionally surpass natural heparin, enabling the design of materials with high specificity, reproducibility, and contextual biological tuning. This makes them one of the most rapidly advancing areas in modern biomaterials chemistry and regenerative medicine. The properties of the most popular heparin mimetics are listed in the Table 9. 

## 5. Mechanisms of Action in *in Vitro* and *in Vivo* Models

Designing polymers with desirable antithrombotic properties requires a thorough understanding of the complex mechanisms of material–blood interactions and effective validation of these interactions in appropriately selected research models. This challenge centers on achieving an optimal balance between hemocompatibility, mechanical durability, and controlled biological activity [263,264].

### 5.1. In Vitro Tests for Evaluating Hemocompatibility and Thrombogenicity of Polymeric Biomaterials

*In vitro* tests constitute the first step in assessing the antithrombotic properties of polymers and include both functional coagulation tests and analyses of material–protein and material–platelet interactions. All tests are conducted in accordance with the international guidelines of ISO 10993-4:2017 [265] and ASTM F756-17 [266], F2150-19 [267], and F2888-13 [268], which define standards for assessing the interaction of materials with blood and indicate reference methods (Table 10). The most commonly used laboratory tests are measurements of prothrombin time and activated partial thromboplastin time. The activated partial thromboplastin time (aPTT) test is one of the fundamental tools used to assess the antithrombotic properties of polymeric materials that come into direct contact with blood. In the activated partial thromboplastin time (aPTT) assay, plasma is first incubated with a surface activator—typically silica, kaolin, or ellagic acid—together with a mixture of phospholipids, known as partial thromboplastin. The addition of calcium ions subsequently initiates the coagulation process, and the time required for clot formation is measured. The aPTT test assesses the functional activity of coagulation factors involved in the intrinsic and common pathways of the coagulation cascade, including factors XII, XI, IX, VIII, X, II, and I. Therefore, it constitutes a preliminary, functional screening test to assess whether a given material induces or inhibits activation of the coagulation system after contact with plasma. For example, surfaces modified with heparin or sulfonated polymers cause a significant prolongation of the aPTT [248,265,269]. The evaluation of polymeric materials intended for contact with blood requires consideration of the prothrombin time (PT) test, which measures the ability of plasma to form a clot following activation of the extrinsic and common pathways of the coagulation system. In the PT test, plasma is incubated with a tissue activator (e.g., thromboplastin) and calcium, and the time to clot formation is recorded [248]. A prolonged PT result in the presence of the tested material indicates that the polymer surface reduces the activation of factor VII or subsequent extrinsic pathway factors (VII, V, X, II, and low I) and may therefore contribute to the antithrombotic effect. In biomaterials research, the PT test serves as an indicator of unfavorable coagulation activation due to contact with blood, and its prolongation is considered a positive characteristic in the context of hemocompatibility [270].

Thrombin time (TT) is a coagulation assay that evaluates the final stage of the coagulation cascade—the conversion of fibrinogen to fibrin catalyzed by thrombin. In this test, a standardized concentration of thrombin is added to citrated plasma that has been in contact with the tested material, and the time required for fibrin clot formation is recorded in seconds. The reference range for TT depends on the thrombin concentration used in the assay. In studies of anticoagulant polymers, the TT test provides information on whether the material or its surface modification interferes with the terminal step of coagulation, i.e., fibrin formation. Materials that release or expose unfractionated heparin-like functional groups, such as sulfate or sulfonate moieties, often lead to a marked prolongation of TT, which indicates effective inhibition of thrombin activity and reduced fibrin generation. Unfractionated heparin markedly prolongs TT because it directly inhibits thrombin activity; however, this assay is not routinely used for therapeutic monitoring. In the context of biomaterial research, a pronounced extension of TT in the presence of a heparin-mimicking polymer indicates that the material effectively interacts with thrombin or impedes fibrin formation. The TT test may also serve as a qualitative tool to confirm the presence of heparin or heparin-like structures, for example, when evaluating potential heparin contamination or assessing the anticoagulant potential of surface-bound functional groups. In contrast, LMWH, Fondaparinux, and direct factor Xa inhibitors typically do not affect TT results, as these agents act predominantly—or exclusively—by inhibiting factor Xa rather than thrombin itself [248].

Because classical coagulation assays (PT, aPTT, TT) primarily assay soluble coagulation pathways and the enzymatic conversion steps of the coagulation cascade, they are necessary but not sufficient to characterize the haemocompatibility of blood-contacting polymers. Effective evaluation, therefore, combines clotting-time assays with targeted assays of material–platelet interactions, including quantitative and qualitative measures of platelet adhesion, markers of platelet activation, and functional aggregation tests performed under both static and flow conditions. Such multi-tiered testing is recommended by current standards for medical-device haemocompatibility testing, which emphasize selection of complementary *in vitro* assays that reflect the intended contact mode (direct/indirect), contact time, and flow environment of the device [98,105]. Experimentally, platelet adhesion is commonly quantified by (i) direct imaging of material surfaces after blood exposure (scanning electron microscopy, fluorescence/confocal microscopy) to enumerate bound platelets and assess morphology, (ii) measurement of residual platelet counts in the bulk blood sample to estimate platelet removal by adhesion, and (iii) biochemical assays that detect platelet-specific released proteins (e.g., platelet factor 4, β-thromboglobulin) or surface activation markers. Platelet activation is profiled by flow cytometry (surface expression of P-selectin/CD62P and activated GPIIb/IIIa), and soluble activation markers. Platelet aggregation, the process of platelets merging into larger clusters or aggregates, is the next step and often depends on prior adhesion and activation. In the context of materials, aggregation can be assessed by the number or size of aggregates formed on the material surface or in the flow containing the material in a flow loop. In studies of antithrombotic polymers, the goal is to demonstrate that their surfaces limit platelet adhesion and activation—which also translates into reduced aggregation—which in turn indicates favorable hemocompatibility. Aggregation and thrombus growth under physiologic shear are especially well assessed in dynamic perfusion systems (parallel-plate or microfluidic flow chambers) that replicate arterial or venous shear rates; such dynamic assays often reveal pro- or anti-thrombotic tendencies that are not apparent in static tests [271,272]. Interpretation of platelet-centric assays requires careful control of pre-analytic variables (anticoagulant choice, platelet count, donor variability), and an integrated readout that considers both the quantity of adhered platelets and their activation state. Importantly, the relationship between the amount of adsorbed fibrinogen and platelet adhesion is not strictly linear: differences in fibrinogen conformation and the accessibility of functional epitopes can result in surfaces with comparable protein loading but divergent platelet responses. Therefore, assessing protein adsorption quality (conformation/epitope exposure) together with platelet assays improves predictive power for thrombogenicity of polymeric surfaces [273,274,275].

An important complementary test is the hemolysis test, which determines whether the material causes damage to erythrocyte membranes. According to ASTM F756-17 [266], a material is considered non-hemolytic if the percentage of hemolysis does not exceed 2%. This assessment is often supplemented with a complement activation test, which measures the levels of proteins such as C3a, C5a, or SC5b-9 using ELISA. An overview of standardized tests for evaluating hemocompatibility and thrombogenicity of polymeric biomaterials is provided in Table 10.

Despite significant progress in the development of polymeric materials with anticoagulant or antithrombogenic properties, their comparative evaluation remains challenging due to the lack of fully standardized hemocompatibility testing methodologies. Commonly employed *in vitro* assays—including protein adsorption, platelet adhesion and activation, coagulation time measurements, and hemolysis—vary considerably with respect to experimental protocols, incubation times, surface-blood contact conditions, and analytical endpoints [276]. Moreover, substantial discrepancies exist regarding the applied shear and flow conditions, with many studies relying on static blood-contact models that insufficiently reflect the complex hemodynamic environment encountered *in vivo*, particularly in cardiovascular applications. The choice of blood source (human versus animal), donor variability, and the type of anticoagulant used (e.g., heparin, citrate, EDTA) further contribute to inter-study variability and can markedly influence the observed blood–material interactions [265,277]. As a consequence, direct quantitative comparison of hemocompatibility data across different polymer systems is often limited, and reported improvements in anticoagulant performance may partially reflect methodological differences rather than intrinsic material properties. This lack of standardization hampers the establishment of robust structure–property relationships and complicates the translation of promising *in vitro* findings into reliable *in vivo* performance. Addressing these challenges will require the development of harmonized testing protocols that better account for physiologically relevant flow conditions and biological variability, in line with existing regulatory frameworks such as ISO 10993-4 [265,278].

### 5.2. In Vivo Models for Evaluating the Hemocompatibility and Thrombogenicity of Polymeric Biomaterials

The *in vivo* evaluation of polymeric materials is a crucial step in determining their thrombogenic potential, particularly for applications involving direct blood contact, such as vascular grafts, stents, heart valves, and extracorporeal devices. While *in vitro* assays provide valuable preliminary information regarding protein adsorption, platelet adhesion, and coagulation activation, *in vivo* models are essential for understanding the complex interactions between the material, blood components, and the dynamic physiological environment.

Several animal models have been developed to assess the thrombogenicity of polymeric biomaterials (Table 11). Among the most widely used are arteriovenous (AV) shunt models, in which the test material is incorporated into a shunt connecting an artery and a vein (typically in rabbits, pigs, or dogs). These models allow continuous blood flow under physiological shear stress, enabling quantitative evaluation of thrombus formation, platelet activation, and fibrin deposition on the material surface. The arterial implantation model, such as placement of polymer-coated stents or vascular grafts in pig or rabbit arteries, provides a clinically relevant environment for high-shear flow and pulsatile conditions typical of human arterial circulation. For materials intended for low-shear or venous applications, venous graft or catheter models are employed. In these models, polymeric tubes or coatings are implanted into veins (e.g., the jugular or femoral vein) to study the propensity for thrombus formation and occlusion. Extracorporeal circulation models in larger animals, such as pigs or sheep, are also valuable for simulating conditions encountered in devices like hemodialyzers or oxygenators. These systems permit real-time monitoring of clot formation, blood flow, and systemic coagulation markers such as thrombin–antithrombin complexes or D-dimer levels. A simpler and less invasive approach involves subcutaneous or intravascular implantation of polymer discs or rods, which, although not fully replicating physiological flow, allow for histological examination of platelet and fibrin deposition, inflammatory response, and tissue integration. These short-term studies are often used as preliminary screening before more complex vascular models.

Key methods for assessing thrombogenicity *in vivo* include macroscopic thrombus quantification (thrombus weight or occlusion percentage), histological and immunohistochemical analyses (platelet and fibrin staining), scanning electron microscopy (SEM) for surface characterization, and measurement of systemic coagulation markers in blood samples. Some studies also use imaging techniques such as angiography or ultrasound to evaluate vessel patency and flow. Despite their value, *in vivo* models have several limitations. Surgical variability, interspecies differences in coagulation systems, and the short duration of experiments may affect reproducibility and translational relevance. Moreover, ethical considerations and cost restrict extensive animal use. Therefore, *in vivo* testing should be carefully integrated with in vitro assays and computational modeling to obtain a comprehensive assessment of material thrombogenicity [257,258].

### 5.3. Structure–Function Relationships in Polymer Materials and Thrombogenicity

**Figure 17:**
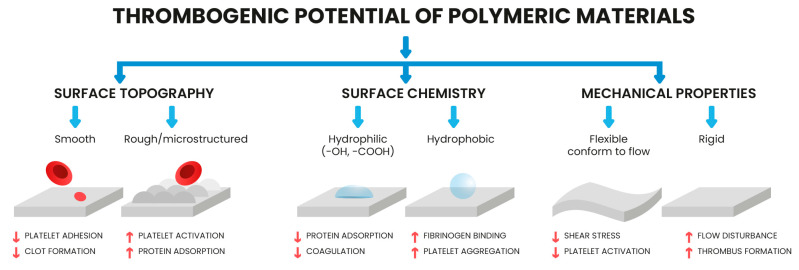
Structural and physicochemical properties affecting the thrombogenic potential of polymeric materials.

The thrombogenic potential of polymeric materials is strongly dependent on their structural and physicochemical properties, which determine how they interact with blood components (Figure 17).

Surface topography is a key factor: smooth polymer surfaces generally demonstrate lower platelet adhesion and reduced clot formation, whereas rough or microstructured surfaces enhance protein adsorption and platelet activation. For example, Liu et al. demonstrated that in PEG functionalized lactide-caprolactone copolymers, the presentation of PEG chains at the surface was directly correlated with reduced platelet adhesion, illustrating the structure-function relationship in hemocompatible polymeric biomaterials [289]. Chemical functionality of the polymer surface also plays a critical role. Hydrophilic groups such as hydroxyl (-OH) or carboxyl (-COOH) moieties can reduce nonspecific protein adsorption, mitigating the initial steps of coagulation, whereas hydrophobic surfaces tend to promote fibronogen binding and platelet aggregation. A recent study of heparin-loaded PEG-based hydrogels applied onto film substrates reported significantly reduced platelet adhesion and prolongation of plasma recalcification time, indicating improved antithrombogenicity [234]. Moreover, polyurethane films chemically decorated with PEG-2000, gelatin, gelatin-aspirin, gelatin-heparin, and combinations thereof showed that the heparin-modified surfaces inhibited platelet adhesion more than aspirin, and PEG itself (as a negative control) showed the lowest platelet adhesion [290].

Mechanical properties further influence thrombogenicity. Polymers with higher flexibility or elasticity, such as silicone elastomers used in catheters, can conform to dynamic vascular environments, minimizing flow disturbances and subsequent platelet activation. In contrast, rigid materials such as polyethylene terephthalate (PET) or Dacron (woven PET grafts) can create areas of high shear stress in the blood flow, which may promote thrombus formation. However, modifying the surface of nonwoven PET fibers with polyethylene glycol (PEG) has been shown to reduce platelet adhesion and activation, bringing thrombogenicity levels closer to those of well-established hemocompatible materials [291]. Additionally, nanostructured surfaces allow selective protein adsorption, enabling design strategies that favor anticoagulant protein binding while inhibiting prothrombotic proteins. For instance, polyurethane urea (PUU) films were textured with submicron patterns and grafted with PEG; this dual strategy (physical topography and chemical grafting) showed much greater reduction in protein adsorption, platelet adhesion/activation, and bacterial adhesion than chemical or topographic treatment alone [28].

These examples underscore the principle that structure dictates function: by carefully tuning topography (smoothness vs. roughness), surface chemistry (hydrophilicity/hydrophobicity, anticoagulant molecule immobilisation), and mechanical characteristics (rigidity vs. flexibility, matching vessel compliance), researchers can design polymeric materials with controlled interactions with blood, optimizing hemocompatibility for vascular and extracorporeal applications. Understanding these relationships is essential for translating *in vitro* findings to *in vivo* performance and ultimately to safe clinical use.

## 6. Clinical and Translational Significance of Antithrombotic and Antifouling Surfaces

The development of antithrombotic coatings used on implants and medical devices—such as vascular grafts, stent-grafts, vascular catheters, valves, vena cava filters, or dialysis membranes—represents a key direction in the advancement of biomaterials. The main goal of these technologies is to reduce thrombus formation on the surface of foreign materials, thereby improving the long-term patency and functionality of devices and decreasing the need for systemic anticoagulant therapy, which carries a risk of bleeding complications [18,292]. Modern strategies include: heparin coatings (chemically bonded to the luminal surface of grafts or dialysis filters); bioactive polymers that modulate blood cell adhesion and activation; drug-eluting coatings releasing antiplatelet or antiproliferative agents; and hybrid solutions combining antithrombotic, anti-inflammatory, and antibacterial properties [293].

In clinical translation, it is essential to maintain a balance between local efficacy (e.g., reducing thrombosis on the surface of a graft) and systemic safety, above all, avoiding an increased risk of bleeding. An additional challenge is the complex regulatory classification—these products are often categorized as medical devices or combination products, which affects market authorization processes and the requirements for preclinical and clinical documentation.

### 6.1. Coatings for Stents, Grafts, Catheters, Valves, and Extracorporeal Systems

Heparin-bonded vascular grafts and stent-grafts represent the most clinically established group of antithrombotic surfaces. In these devices, heparin is covalently immobilized on the luminal surface of a polymer, such as expanded PTFE, while maintaining its biological activity (for example, the CBAS Heparin Surface). The GORE PROPATEN Vascular Graft, which features heparin-bonded ePTFE, has demonstrated improved patency rates compared to non-coated ePTFE grafts in peripheral bypass surgery. This has resulted in reduced early thrombosis and patency rates that are comparable to those of autologous vein grafts [293,294]. The randomized studies have compared PROPATEN with standard ePTFE, and the results were published on ClinicalTrials.gov [295]. These trials emphasize the significance of formal clinical trial frameworks in the translational evaluation of these coatings, ensuring a thorough assessment, even in light of potential gaps in the available public datasets [295,296]. The same heparin-bonding technology has been extended to other devices, including grafts and stent-grafts for hemodialysis access and peripheral or thoracic endovascular procedures (e.g., GORE VIABAHN Endoprosthesis with CBAS Heparin Surface). Clinical data indicate reduced early thrombotic events and prolonged primary patency of vascular access compared to non-coated controls, which is particularly relevant in patients with limited options for native fistulas [297,298]. Surface-modified stents and flow diverters are making significant strides in neurovascular interventions. Innovative devices like the FRED X employ cutting-edge “antithrombotic surface treatment” technologies that not only minimize platelet adhesion but also enhance the potential for rapid endothelialization. Early clinical evaluations reveal a compelling safety profile alongside sustained aneurysm occlusion rates. This promising evidence demonstrates that surface engineering can skillfully navigate the intricate balance between thrombotic and hemorrhagic risks in intracranial procedures, ultimately improving patient outcomes [298,299].

In hemodialysis, “immediate-access” AV grafts such as Flixene represent another important translational example. These multilayer synthetic grafts, designed with tailored wall architecture and surface modifications, can be cannulated within 24–72 h after implantation, reducing the need for temporary central venous catheters. Cohort studies report acceptable long-term patency and complication profiles, which translates into more continuous dialysis treatment and fewer catheter-related complications in routine practice [300,301,302]. Coronary stents provide another relevant arena. The COBRA PzF stent uses a very thin fluoropolymer (poly-hexafluoropropylene, PzF) coating that improves thromboresistance and is specifically designed to permit shortened dual antiplatelet therapy (DAPT) in patients at high bleeding risk. Clinical studies and registries suggest that PzF-coated platforms may allow for abbreviated DAPT without a prohibitive increase in stent thrombosis, positioning surface chemistry as a key determinant of antithrombotic therapy duration in vulnerable patient subgroups [132,303].

Advances in medical devices are also being made through antithrombotic and antifouling modifications to intravenous catheters [304], oxygenators, ECMO circuits [305], centrifugal pumps [306,307], and dialyzers [308,309,310]. New clinical trials are being conducted to use new solutions using special coatings on medical devices and tools with the use of special premedications in the fight against thrombosis [311,312]. In addition to studies confirming the clinical usefulness of the developed antithrombogenic coatings, new, promising solutions are being introduced. For example, polydopamine-based methods for immobilizing antithrombotic molecules are the subject of patent applications and preclinical studies, aiming to create versatile platforms for surface modification of biomaterials to provide safer and more effective healthcare solutions [313]. Despite ongoing extensive research, many of these concepts remain in preclinical or early clinical development, highlighting the direction towards modular, “plug-and-play” surface functionalization of various blood-contacting devices.

### 6.2. Drug-Delivery Systems for Local and Controlled Anticoagulant Release

The concept of local, controlled release of anticoagulants from vascular implants and devices—such as stents, grafts, catheters, or filters—represents one of the most promising directions in the development of modern biomaterials. The goal of these technologies is to achieve a strong, localized antithrombotic effect on the device surface while minimizing systemic exposure, which should reduce the risk of systemic bleeding. Historically, the first innovations in this area came from interventional cardiology, where drug-eluting stents (DES) releasing antiproliferative agents predominated. Current development is moving toward coatings that combine antiproliferative activity with the release of antithrombotic substances, and even precisely designed drug combinations that modulate different stages of the coagulation cascade [314].

Modern drug-delivery systems rely on advanced coating-design strategies that provide defined release kinetics and pharmacological stability. These include: (1) biodegradable polymers (e.g., PLGA, PLA, PCL), which gradually degrade and enable sustained release of active substances [315]; (2) hydrogel coatings, which enhance retention of hydrophilic drugs and improve the stability of heparin or its analogues [132]; (3) multilayer systems, in which one layer provides immediate release while subsequent layers ensure prolonged activity [132]; (4) nanostructured carriers, such as polymeric or liposomal nanoparticles embedded within the coating matrix, allowing pulsatile or directional drug release [316]; (5) hybrid coatings, combining antiproliferative properties (e.g., sirolimus, paclitaxel) with coagulation modulation (e.g., heparin, thrombin analogues, factor Xa inhibitors) [317]. Such solutions enable the development of implants that simultaneously exhibit bioactivity, long-term hemocompatibility, and controlled drug release, significantly enhancing their clinical potential compared with traditional passive biomaterial coatings [318].

Current drug-eluting stents (DES) used in clinical practice primarily emphasize antiproliferative drugs like sirolimus, everolimus, and zotarolimus to effectively prevent neointimal hyperplasia and restenosis. However, the potential for local delivery of direct antithrombotic agents such as heparin, fondaparinux, and direct thrombin or factor Xa inhibitors from stent surfaces is still largely under exploration. Increasing evidence from preclinical and early translational studies highlights the necessity for an “ideal” stent that not only combats restenosis but also modulates local coagulation and platelet activation, while facilitating rapid endothelial recovery. Embracing this approach could revolutionize stent technology and improve patient outcomes significantly [293,319]. This has stimulated the development of intelligent, stimulus-responsive coatings capable of releasing anticoagulants in response to specific biochemical cues, such as elevated thrombin activity or oxidative stress. In current clinical practice, the closest approximation to a “built-in” antithrombotic surface without classic burst-type drug release is represented by fluoropolymer-coated platforms (e.g., PzF), which exhibit improved thromboresistance and may safely permit shorter DAPT durations in selected patients [320]. At the same time, drug-coated balloons (DCB) are being tested in populations in whom standard antithrombotic regimens are challenging, including those at very high bleeding risk or with contraindications to prolonged DAPT [293,297,321].

Clinical trial registries contain numerous studies that combine endovascular interventions with optimized antithrombotic regimens—from trials comparing different DES/DCB strategies to those tailoring DAPT length or anticoagulation type according to device characteristics and bleeding risk. In many of these protocols, the surface chemistry of the implanted device (e.g., polymer-free vs. durable polymer, heparin-bonded vs. non-coated, PzF vs. conventional fluoropolymer) is explicitly taken into account in the design of the antithrombotic strategy, underscoring the tight interplay between material science and pharmacotherapy in translational cardiovascular medicine. The study NCT05033964 [298] (The DESyne BDS Plus RCT: A Randomized Clinical Trial to Assess the Elixir DESyne BDS Plus Drug Eluting Coronary Stent System for the Treatment of de Novo Native Coronary Artery Lesions) is a prospective, multicenter, randomized, single-blinded clinical trial designed to evaluate the safety and effectiveness of the new DESyne BDS Plus coronary stent system—a device that simultaneously releases three agents: sirolimus, rivaroxaban, and argatroban. This stent is engineered as a system combining antiproliferative and antithrombotic activity, making it one of the most technologically advanced solutions in the field of personalized drug-elution strategies. A total of 200 patients with native, de novo coronary artery lesions will be enrolled. In a subset of patients, OCT imaging (approximately 60 individuals) will be performed, and another subgroup will participate in a pharmacokinetic (PK) substudy evaluating the dynamics of release of the three drugs over the first 7 days after implantation. Clinical follow-up will extend to 3 years.

The study NCT04814212 [299] (Drug-Coated Balloon in Anticoagulated and Bleeding Risk Patients Undergoing PCI) evaluates a coronary angioplasty strategy in patients with chronic coronary artery disease (CAD) or acute coronary syndromes (ACS) who are at high risk of bleeding. Two strategies are compared: (1) DCB + shortened DAPT duration; (2) DES + standard DAPT duration. Drug-coated balloons (DCB), particularly those coated with paclitaxel, represent an attractive alternative to DES—especially in patients for whom a permanent implant increases the risk of bleeding complications or makes it difficult to shorten antiplatelet therapy. The study hypothesis assumes non-inferiority of DCB compared with DES, followed—if confirmed—by an assessment of the potential superiority of a no-implant strategy. This trial represents a paradigm shift compared with classical DES. It is based on the absence of a permanent implant and on local, short-term drug release from the balloon surface, which markedly reduces the risk of late thrombosis and bleeding associated with prolonged antiplatelet therapy.

The concept of controlled, local anticoagulant release represents an important and rapidly developing direction in vascular implant technology. Early clinical evidence—including the DESyne BDS Plus trial—confirms the feasibility and safety of devices that combine antiproliferative effects with local modulation of coagulation. However, a full understanding of their safety profile requires further studies involving larger populations and longer follow-up, with particular attention to bleeding risk, immunogenicity (e.g., HIT), late thrombosis, and interactions between drugs released from the coatings [320].

A particularly interesting translational example at the drug level is anfibatide, a snake-venom-derived peptide that targets platelet GPIbα and selectively inhibits von Willebrand factor–mediated platelet adhesion and aggregation [322]. Preclinical studies showed effective inhibition of thrombus formation with minimal prolongation of bleeding time. In a phase I randomized clinical trial in healthy volunteers, anfibatide reduced VWF-dependent platelet aggregation without clinically relevant bleeding or immune reactions. Although currently investigated as a systemically administered agent, its highly specific mechanism suggests potential future applications as a surface-bound antithrombotic ligand, illustrating how drug development pipelines and surface-engineering strategies can converge at the translational interface.

### 6.3. Regulatory and Safety Challenges: Bleeding, Immunogenicity, and Evidence Requirements

The introduction of new surface materials and local anticoagulant-release systems requires simultaneous consideration of safety aspects and regulatory rigour that determine their clinical applicability. A core premise of local drug-delivery technologies is to achieve a strong local effect while maintaining minimal systemic exposure, which theoretically reduces the risk of bleeding. However, the safety profile of these solutions is multidimensional and requires detailed preclinical and clinical evaluation [317,323].

The major risks associated with implants releasing anticoagulants include: (1) Increased systemic exposure to anticoagulants; (2) Immunogenicity of coatings containing heparin or heparin-mimetic substances; and (3) Local coating toxicity and its impact on healing processes [323].

**Figure 18:**
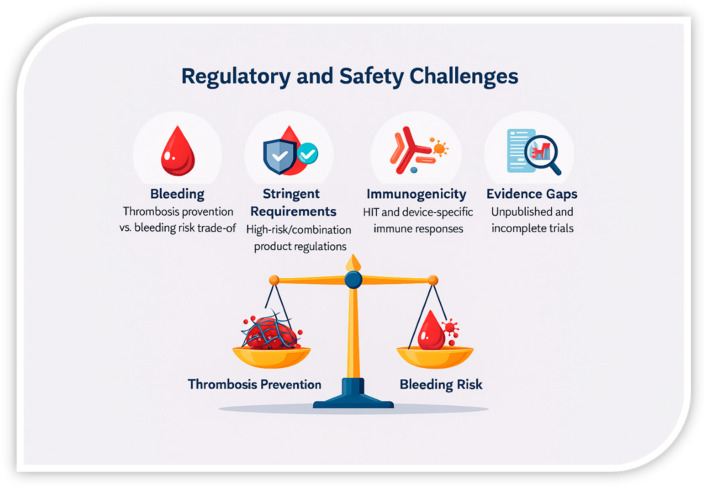
Regulatory and safety challenges for antithrombotic and antifouling surfaces. Schematic representation of the key challenges limiting clinical adoption of antithrombotic and antifouling coatings: the trade-off between thrombosis prevention and bleeding risk, stringent high-risk/combination-product regulatory requirements, the potential for immunogenicity (including HIT and device-specific immune responses), and persistent evidence gaps due to unpublished or incomplete clinical trials.

The clinical adoption of antithrombotic and antifouling surfaces presents several critical challenges that must be addressed (see Figure 18). Many of these devices are categorized as high-risk products (e.g., Class III) and are often classified as combination products, integrating drugs and devices. Consequently, they must adhere to rigorous standards for hemocompatibility, thrombogenicity, coating stability, and long-term clinical efficacy. Regulatory guidance and expert consensus on evaluating coronary and peripheral stents in Europe highlight the urgent need for thorough preclinical testing and randomized clinical trials with definitive clinical endpoints before any new coatings can be integrated into standard medical practice. Addressing these challenges is essential for ensuring patient safety and advancing medical technology [324,325,326].

The fundamental trade-off between thrombosis prevention and bleeding risk remains central. Contemporary guidelines and position papers from cardiovascular societies emphasize individualized antithrombotic therapy based on device type, ischemic risk and bleeding risk [327]. For surfaces labeled as “highly thromboresistant,” which support shorter or less intensive antiplatelet and anticoagulant therapies, it is essential to demonstrate low device-related thrombosis rates without increasing major bleeding. This requires robust clinical trials with extended follow-up and diligent post-marketing surveillance to track rare but serious events. Ensuring safety and efficacy is crucial for building trust and achieving optimal patient outcomes [328,329,330].

Immunogenicity and device-specific complications are critical concerns that cannot be overlooked. Heparin-bonded surfaces offer significant benefits for graft patency; however, they may pose serious risks for patients with a history of heparin-induced thrombocytopenia (HIT) [322,329]. Even minimal exposure to surface-bound heparin could potentially provoke serious immune reactions. Therefore, it is essential to exercise caution and consider alternative materials for this vulnerable subgroup, as the available observational data strongly support the necessity of tailored approaches to enhance patient safety and outcomes [331,332]. Fortunately, in contrast, initial clinical experience with agents like anfibatide indicates that bio-derived antiplatelet molecules can, if properly engineered, display a favorable immunological profile without detectable antibody formation; however, larger and longer studies are necessary to confirm this [333,334]. Improper drug-release kinetics or premature degradation of the coating may lead to excessive concentrations of active substances in the bloodstream, increasing the risk of bleeding. For this reason, pharmacokinetic (PK) studies and assessment of subtherapeutic concentrations are required components of clinical trial designs. An example is the previously cited NCT05033964 [298] (The DESyne BDS Plus RCT), which included a dedicated PK substudy evaluating sirolimus, rivaroxaban, and argatroban concentrations up to 7 days after implantation. The results indicated that systemic anticoagulant levels remained low, correlating with a low incidence of bleeding complications during follow-up.

Heparin—whether covalently bound or immobilized on a surface—can form complexes with Platelet Factor 4 (PF4), which in some cases leads to the development of anti-PF4/heparin antibodies. This does not always result in clinical HIT, but it requires monitoring. A translational example is the study NCT02165761 [335] (Evaluation of Anti-platelet Factor 4/Heparin Antibodies in Hemodialysis Patients Implanted With the GORE^®^ Hybrid Vascular Graft Versus Non-heparin Bonded Synthetic Vascular Grafts), which assessed seroconversion of anti-PF4/H antibodies in dialysis patients implanted with heparin-bonded grafts versus non-coated synthetic grafts. The study included a schedule of serial immunological measurements (D7–12M) and correlation with clinical parameters (platelet counts, thrombotic events, graft patency). These findings highlight that immunogenicity assessment is a critical element of the safety evaluation of modern bioactive coatings.

In the study NCT04814212 [299] (Drug-Coated Balloon in Anticoagulated and Bleeding Risk Patients Undergoing PCI), the PCI strategy using a drug-coated balloon (DCB) was compared with a conventional drug-eluting stent (DES) in patients with coronary artery disease (stable CAD or ACS) who were at high bleeding risk (HBR) or required long-term anticoagulation. Although a DCB is not an implant with a permanent anticoagulant-eluting coating, the DEBATE trial is important as a model of a strategy aiming to minimize antiplatelet therapy in HBR patients, which represents an analogous effort to reduce systemic exposure and minimize bleeding, similar to the rationale behind implants with local drug release.

The study demonstrates that when systemic antiplatelet/anticoagulant therapy must be reduced, clinical trials must include clearly defined bleeding criteria, the ability to compare strategies reducing systemic exposure, and a structured plan for monitoring complications. The study NCT02717039 [336] (Pharmacogenomics of Heparin-Induced Thrombocytopenia) demonstrates that a genetic predisposition to immunological reactions to heparin does indeed exist, which has direct implications for the design and regulatory evaluation of heparin-containing coatings. The risk of HIT can vary substantially across the population and is not predictable without genetic or immunological testing. This means that for heparin-based coatings, immunomonitoring and, optionally, genotyping may become essential components of safety planning and risk-mitigation strategies. At the same time, the study shows that even with well-designed protocols, natural biological variability and genetic susceptibility pose significant regulatory challenges. NCT02717039 illustrates that the immunological risks associated with heparin are real, heterogeneous, and patient-dependent—which implies a need to incorporate immunological monitoring (and potentially genotyping) into protocols for implants and coatings containing heparin.

Gaps and fragmentation in the evidence base present significant challenges for both regulatory bodies and the scientific community. Even for commonly used commercial devices, some critical trials remain unpublished or lack complete reporting. This limitation hinders the ability to conduct high-quality meta-analyses or to make thorough comparisons between various surface technologies. One example is the NCT00205790 trial, which studied heparin-bonded grafts versus standard ePTFE grafts [337], illustrating how incomplete public reporting can persist despite device commercialization. The consistent and systematic use of clinical trial registries, standardized reporting, and multicenter collaborative studies is crucial for the evaluation of new antithrombotic and antifouling materials. By adhering to these high-quality evidence standards, we can ensure that their true clinical value—both in terms of efficacy and safety—is thoroughly documented and understood. This commitment to rigorous evaluation is vital for advancing patient care and safety. A summary, brief description of selected clinical and translational applications of antithrombotic/antifouling coatings is presented in Table 12.

## 7. New Trends and Future Directions in Research

Blood does not tolerate compromise. Surfaces in contact with blood must be hemocompatible and antithrombotic to prevent coagulation activation, inflammation, hemolysis, and thrombus formation, ensuring patient safety and proper device function. Any surface that even briefly disturbs its equilibrium is immediately “read” by the coagulation and immune systems. We believe that the future of antithrombotic materials lies not in passive, smooth surfaces, but in dynamic systems that can identify threats and respond precisely when and where intervention is necessary. Bioactive, signal-responsive materials are at the forefront of this innovative development. Rather than relying on continuous, systemic anticoagulation, these advanced materials can function like a “micro-pharmacy,” effectively storing an inhibitor that is released only upon detecting specific triggers—such as elevated thrombin levels, increased reactive oxygen species (ROS), or variations in temperature or pH. This tailored approach could significantly enhance patient safety and treatment efficacy [343]. Advanced intelligent systems are revolutionizing medical technology by enabling selective and precisely timed activation. One compelling example is thrombin-responsive coatings, which degrade or disassemble the polymer matrix in response to the coagulation enzyme thrombin. This innovation facilitates the targeted release of anticoagulants—such as heparin or hirudin—exactly where and when thrombus formation occurs, substantially minimizing the risk of systemic bleeding. Similarly, pH-dependent systems activate in the acidic environments of inflamed tissues or tumors, and redox-sensitive materials undergo degradation in response to elevated intracellular glutathione levels. Additionally, enzyme-responsive materials that react to metalloproteinases or esterases released during pathological processes represent a rapidly growing area of research. Applying these coatings to stents, vascular implants, or bioactive dressings marks a significant step toward personalized therapy. This means that the materials not only fulfill a mechanical role but also actively engage in biological processes. By supporting healing and minimizing complications, these intelligent systems are truly transforming patient care [344].

The second area of progress in this research focuses on strategies that modulate the immune response and hemostasis. Instead of merely reducing protein adsorption and the activation of factor XII, new coatings aim to “reprogram” the interactions at the blood-material interface. For example, polymer coatings with “hidden positive charges” contain positively charged segments that strongly bind to factor XII, but these segments are partially shielded by hydrophilic areas, such as polyethylene glycol (PEG). This design allows for strong but “safe” binding of factor XII, limiting its ability to propagate the signal and generate thrombin while still maintaining overall hemostatic function. In flow models and an arteriovenous shunt model, this approach demonstrated a reduction in thrombin generation without extending clinically relevant clotting times [344,345]. Selective antifouling coatings are currently being developed that serve two main purposes. First, they create superhydrophilic, zwitterionic surfaces that minimize the adsorption of fibrinogen and the adhesion of platelets. Second, these coatings include anchored adhesive motifs, such as peptide sequences and extracellular matrix (ECM) fragments, that promote the attachment of endothelial cells. This dual approach allows for the suppression of unwanted interactions with blood components while simultaneously facilitating the re-endothelialization of implants. Recent studies have shown that densely packed zwitterionic brushes and superhydrophilic phosphorylcholine-based coatings can significantly reduce fibrinogen deposition and thrombus formation in both *in vitro* and *ex vivo* environments, all while ensuring effective colonization by endothelial cells.

A third key direction is multifunctional engineering—combining antithrombotic activity with antibacterial effects. Coagulation reactions and inflammation after biomaterial contact are tightly interconnected; exacerbated inflammation and coagulation can, among other things, lead to a high risk of thrombosis. Heparin-based coatings conjugated with antibacterial components or zwitterionic hydrophilic surfaces simultaneously reduce blood protein adhesion and bacterial colonization [346,347]. However, coating medical materials and devices is often a complex procedure, and in practice, the effect is sometimes unsatisfactory, hindering clinical translation. The work of Q. Shi and Y. Wang’s groups fits perfectly into this trend: heparinized, self-healing polymer coatings with modulation of inflammation. These coatings exhibited long-term stability and effective inhibition of thrombosis while maintaining self-healing capacity. Additionally, they were responsive to ROS and enabled temperature-dependent aspirin release [348]. Another example is anticoagulant coatings with thrombin-responsive nanogels containing components with both antithrombotic and anti-inflammatory activity. *In vitro* and *in vivo* studies confirmed the efficacy and versatility of these coatings on bioprosthetic valves and vascular stents. This modification was characterized above all by improved hemocompatibility, more favorable host response, enhanced re-endothelializatinand antihyperplastic activity [343]. Such integrated “3-in-1” therapies open new possibilities for treatment. Personalization of materials is also emerging on the horizon. Thanks to 3D printing technologies, it is possible to tailor the geometry and coating of a stent to the individual characteristics of a patient. This enables the design of bioresorbable constructs with degradation rates adjusted to healing processes and to the patient’s thrombotic–hemorrhagic risk profile [349].

In the coming years, we can likely expect integration of these technologies with “digital twin” models of the circulatory system, allowing virtual testing of different stent geometries, coating thicknesses and compositions, or release strategies (continuous vs. stimuli-triggered) before the device reaches the patient. Despite promising results, several open questions remain:

- How to ensure scalable, reproducible, and regulatorily acceptable coating technologies for complex, multicomponent responsive systems;
- How to predict the behavior of such materials over the long term (years), taking into account mechanical wear, variability in blood biochemistry, and combination therapies;
- How to combine intelligent antithrombotic coatings with systemic treatment to exploit their synergistic effects [53].

The future of antithrombotic materials, therefore, lies not only in ever “better coatings” or sophisticated systems, but in holistic integration: intelligent, multifunctional interfaces + personalized constructs + digital therapy planning and rigorous clinical validation. Materials, drugs, release mode, and treatment plan must be designed as a single coherent system, rather than as separate building blocks.

## 8. Review Methodology

The main goal of the review was to identify and organize the literature on the synthesis and application of biocompatible polymers with documented antithrombotic properties. After analyzing and selecting the obtained data, we provided extensive information on the achievements in the research area under consideration. We believe that in this way, we have provided the reader with a relatively broad perspective on achievements in the field of thrombosis treatment using special polymers, as well as understanding the emerging trends in this field of research and the prospects for new biomedical applications. The resulting literature review should answer questions related to the development of new research, “outlier” directions, and also the opportunities for implementing the results into clinical practice.

To achieve this objective, a broad literature search was undertaken, using databases typical of multidisciplinary research, such as PubMed, Scopus, HubMed, JURN, and Google Scholar, as well as databases specializing in studies related to medical research, including clinical trials; Cochrane Library, ClinicalTrials.gov, and EU Clinical Trials Register. The considered set of works, including experimental works, was supplemented by items found on websites using the Google search engine. The following keywords were used: “antithrombotic polymer,” “antithrombotic layer,” “heparinized polymer,” “anticoagulant coatings,” “antifouling biomaterials,” “platelet adhesion polymer,” “blood-contacting materials,” in several cases additionally combined with: “surface modification,” “grafting,” “nanofibers,” “controlled release.” In the case of data presented in the chapters presented the introduction and description of the physiological basics of coagulation and antithrombotic mechanisms, or measurements of thrombosis, other keywords were used, such as: “coagulation cascade,” “hemostasis,” “thrombosis mechanism,” “thrombus formation,” “platelet activation,” “platelet aggregation”, “fibrin formation”, “fibrin polymerization” and “analysis thrombosis”, “standard regulatory + analysis thrombosis”. For this review, a systematic search of clinical and preclinical research reports was conducted using combinations of keywords related to antithrombogenic coating technologies, such as: “antithrombogenic coating,” “heparin-bonded graft,” “heparin-coated device,” “drug-eluting + antithrombotic,” “stent-graft + heparin,” “dialyzer heparin coating,” and “vascular implant surface modification.” The studies were evaluated in terms of clinical relevance, translational potential, device classification, and availability of outcome data.

During the search, we focused on works published in the last 15 years as priorities, but we also included classic/groundbreaking works older than 15 years. From the identified records, those most useful for contemporary surface-modification strategies were selected—in particular, studies assessing heparin coatings applied in vascular grafts, stent-grafts, and extracorporeal circulation systems. The initial number of records for analysis from interdisciplinary databases was over 700 entries from each (publications no older than 15 years), most of which were duplicates. The list of clinical and preclinical research reports (Adaptive Clinical Trial, Clinical Study, Clinical Trial, Randomised Controlled Trial) was significantly smaller and did not exceed 50 entries. After reviewing the obtained data, a study was conducted on the research identified as relevant to the assigned and presented topic, and meeting the inclusion criteria. Works that were irrelevant, of low quality, or that did not present a complete set of confirming data were excluded. Most studies and trial descriptions concerning antithrombotic therapy were also discarded, as they mainly referred only to the course of chemotherapeutic treatment. Through this process, 379 items were selected and included in the development of the presented review paper.

## 9. Summary and Conclusions

This comprehensive review of thrombosis research—encompassing its mechanisms, prevention, and treatment—leads to several key conclusions. Despite significant advances in understanding the disease, thrombosis remains a major clinical challenge and a leading cause of global mortality, largely due to still encountered limitations in both basic and clinical research.

Assessing the efficacy of antithrombotic agents remains problematic. Significant challenges arise when accurately determining anticoagulant activity and, more critically, benchmarking their effects. The literature reveals that laboratories often employ divergent *in vitro* testing protocols, making meaningful comparisons nearly impossible. This issue stems from a lack of standardized hemocompatibility testing and the inherent limitations of *in vitro* models in replicating physiological conditions. Specifically, static laboratory environments fail to account for dynamic *in vivo* parameters, such as shear stress and blood flow dynamics—discrepancies that are particularly pronounced in the study of blood vessel implants [350]. Furthermore, maintaining the assumed standard measurement conditions in practice is often difficult [351,352]. Consequently, many materials that perform well under laboratory conditions may ultimately fail in clinical trials. These well-recognized challenges are widely discussed in both academic literature and regulatory analyses [353,354,355]. Today, in addition to various heparin derivatives, the pharmaceutical market offers a wide range of new antithrombotic agents with diverse modes of action, including antiplatelet, anticoagulant, and thrombolytic (fibrinolytic) pathways. These therapies can often replace previously widespread heparin use, allowing treatment to be tailored to individual patients and reducing many of the serious side effects associated with this polysaccharide. However, these new drugs are used primarily as adjunct therapies to prevent thrombosis following surgical or implant procedures. There is still no agent that can inhibit coagulation through mechanisms analogous to those employed by the endothelium—an approach that would likely be significantly safer for patients. Achieving this will require the development of new high-molecular-weight materials specifically synthesized for such applications.

Unfortunately, research on novel synthetic polymers with documented antithrombogenic properties remains relatively limited. Current efforts focus mainly on heparin and its chemical modifications, while much less attention is devoted to the effective modification of other polysaccharides to reduce heparin-related side effects or to identify suitable alternatives.

Contempt extensive research aimed at developing effective antithrombotic surfaces, their practical application in medical devices and implants designed for prolonged blood contact remains limited and far from meeting clinical needs. This is particularly evident in the case of vascular stents, which typically lack such protective coatings—even though clinical and hospital data clearly indicate a postoperative risk of impaired blood flow due to early or late thrombosis. Notable progress has been made in the use of antithrombotic coatings in ECMO (Extracorporeal Membrane Oxygenation) systems, where the latest models incorporate these surfaces not only in the oxygenator but also in the pump and tubing. However, comparing the volume of research on thrombosis prevention through the development of new coatings or antithrombotic materials with the more than tenfold lower number of clinical trials investigating new techniques, materials, and devices highlights the substantial challenges in translating laboratory findings into clinical practice. As a result, only relatively simple coatings with a single functional property have become widely adopted in clinical applications. The primary barrier is that many advanced coatings demonstrate excellent performance in preclinical studies, but their safety—including the biocompatibility of the material and the long-term stability of the coating—requires extensive evaluation, which complicates and delays implementation.

The clinical gap in this field arises primarily from the difficulty in achieving durable and effective antithrombotic performance under physiological conditions. Unlike controlled laboratory environments, *in vivo* conditions introduce unpredictable variables—including inflammation, microbial activity, and mechanical strain—that compromise coating integrity [344,356]. Additionally, the translatability of *ex vivo* animal models is limited by interspecific variability and the difficulty of correlating morphological changes with disease etiology. The lack of standardized protocols for aging studies further complicates the comparative analysis of commercial materials. Therefore, future advancements must focus on increasing material safety, enhancing coating stability, and developing a unified framework for the evaluation of antithrombotic surfaces.

Because the mechanisms underlying thrombosis development and progression are highly complex and influenced by numerous factors—such as platelet activation, the involvement of blood coagulation proteins, and the presence of inflammatory cells—long-term therapy based solely on anticoagulants can significantly affect clinical outcomes. For this reason, combining multiple strategies to create antithrombotic surface layers is essential. An important future direction is the development of materials that are hydrophilic, capable of scavenging reactive oxygen species (ROS), and able to regulate the controlled growth of various cell types.

The use of specialized polymers may not only provide antithrombotic properties but also enable the integration of additional functions crucial to clinical performance, such as anti-inflammatory and antibacterial activity. Beyond expanding the practical applicability of current findings, future efforts should focus on creating next-generation surfaces inspired by the endothelial glycocalyx or on developing slippery, liquid-infused porous surfaces (SLIPS) that behave similarly to natural neointima. These technologies are likely to result in hybrid materials—polymer matrices combined with endothelial cell layers—which appear particularly promising for vascular grafts and bioresorbable stents.

The next major direction involves designing pH- or enzyme-responsive coatings that release antithrombotic agents only when needed. Consequently, the development of sophisticated controlled-release systems based on polymer–antithrombotic conjugates is crucial. Such systems may incorporate nitric oxide (NO) donors, heparin, direct thrombin inhibitors (DTIs), or factor Xa inhibitors. Of particular importance is the localized release of antithrombotic substances directly from device surfaces (e.g., stents, grafts, catheters) to prevent thrombosis without inducing systemic effects, using self-renewing or sustained-release mechanisms. Thus, new polymers are needed that degrade in concert with vascular healing. Over the coming decade, such materials are expected to become standard components of blood-contacting devices, substantially reducing thrombosis risk and decreasing reliance on systemic anticoagulants.

Studies introducing new materials or coatings must be governed by the fundamental medical principle: primum non nocere (first, do no harm). The design of antithrombotic surfaces requires a critical balance between efficacy and safety; excessive inhibition of thrombin or platelet activation inherently increases the risk of haemorrhage. A significant hurdle remains the aforementioned methodological gaps in hemocompatibility testing. Furthermore, many studies overlook the biological complexity of physiological thrombosis by focusing solely on platelets and fibrin. In reality, the complement system, leukocytes, neutrophil extracellular traps (NETs), macrovesicles, and inflammatory mediators play decisive roles in pathogenesis and clinical outcomes. Paradoxically, ‘antifouling’ strategies can be overly effective; by limiting protein adsorption and cell adhesion, they may simultaneously impede re-endothelialisation and vascular healing, potentially increasing long-term complications. Another critical issue is the localization of the effect and the phenomenon of ‘leaching.’ While many systems rely on the controlled release of active substances (e.g., heparin or nitric oxide), they often lack a rigorous mass balance analysis, a defined release profile under dynamic flow, and a systemic bleeding risk assessment during prolonged micro-release. Conversely, ‘immobilized’ coatings offer superior chemical stability but suffer from reduced bioactivity over time due to protein masking of active sites and material ageing. When conducting research, it is crucial to recognize the limitations of animal models in preclinical *in vivo* studies. These constraints extend beyond species-specific differences to include brief observation periods, low statistical power, and, consequently, limited translational relevance to clinical practice. Short-term studies lasting only hours or days fail to address clinical questions that require months or years of observation. In current clinical practice, patients receiving implants with antithrombotic components still undergo standard treatment (DAPT or anticoagulants, often at slightly reduced doses). Therefore, investigating the interactions between biomaterial coatings and concurrent therapies is critical. Many studies fail to evaluate whether a coating permits safe dose reductions, which would offer a significant clinical benefit, or whether it exerts additive effects that increase bleeding risks or lead to dangerous interactions (e.g., involving nitric oxide systems and antiplatelet drugs).

## Figures and Tables

**Table 1:** Examples of antithrombotic drugs with antiplatelet and anticoagulation or thrombolytic (fibrinolytic) mechanisms of action.

Action	Name of Drug	Target
antiplatelet	aspirin	COX-1
	clopidogrel	P2Y12
	prasugrel	
	ticagrelor	
	cangrelor	
	cilostazol	PDE
	dipyridamole	
	vorapaxar	PAR1
	baciximab	αIIbβ3
	eptifibatide	
	tirofiban	
anticoagulation	warfarin	Vitamin K Cycle
	heparins	Factor Xa, Thrombin (through binding antithrombin)
	danaparoid	
	fondaparinux	Factor Xa
	rivaroxaban	
	apixaban	
	edoxaban	
	betrixaban	
	hirudins	Thrombin
	argatroban	
	dabigatran	
thrombolytic (fibrinolytic)	streptokinase	Non-fibrin-specific
	urokinase	
	anistreplase (APSAC)	
	alteplase	Fibrin-specific
	reteplase	
	tenecteplase	
	prourokinase	

COX-1—cyclooxygenase-1; PDE—phosphodiesterase; P2Y12—receptor on platelets involved in their activation and aggregation; PAR1—protease-activated receptor; αIIbβ3—the main membrane glycoprotein receptor on the surface of platelets; Xa—the active form of factor X.

**Table 2:** Review of crucial natural polymers with antithrombotic effect.

Refs.	Polymer	Antithrombotic Activity	Coagulation Mechanism
[80]	Chemoenzymatic heparin analogue “Super 12-mer.”	Anti-FXa IC_50_ ≈ 100 nM; prolonged thrombin lag time; Enoxaparin-like thrombin generation profile; dose-dependent prolongation of clotting parameters (R, α-angle, MA)	FXa inhibition via ATIII; reversible with protamine
[81]	Heparin-mimetic sodium alginate (HepLBm)	*In vivo*: non-coagulable aPTT > 5 μg/100 μL (>600 s), TT > 2.5 μg/100 μL: (>240 s); PT comparable to heparin; reduced PF4 and TAT better than heparin; In vitro: aPTT, TT, PT close or similar to heparin	ATIII-mediated thrombin inhibition
[82]	Photoswitchable arylazopyrazole-modified heparins	*In vitro*: *In vitro*, aPTT significantly reduced the effect of modified UFH, whereas modified enoxaparin showed only a slight difference from the control.	ATIII-dependent, conformation-controlled inhibition
[83]	2-O, 3-O desulfated heparin (ODSH)	Minimal anti-Xa and aPTT effects at low doses; weak anticoagulation at >16 µg/mL	Inhibition of PF4–platelet and PF4–heparin complex formation
[84]	LMWH–octadecylamine conjugate (LMHO)	Prolonged *in vivo* clotting time vs. LMWH; ~97% retained anti-Xa activity; strong albumin binding.	Extended circulation time via albumin binding
[81]	Heparin-mimicking polyurethane	aPTT and TT up to 3× longer vs. PES membranes; dose-dependent surface anticoagulation	Heparin-mimetic surface effects; reduced protein adsorption
[85]	Sulfated chiro-inositol (SCI)	FXa IC_50_ ≈ 0.28 μmol/L with an efficacy of 100% aPTT 32.2 μmol/L, PT 150 μmol/L. selective aPTT prolongation	Allosteric inhibition of FXIa
[86]	Chemoenzymatic heparin oligosaccharides	*In vitro* anti-Xa IC_50_ 2.8–3.6 nM (comparable to Fondaparinux)	ATIII-mediated FXa inhibition
[87,88,89,90]	Sulfated chitosan (various marine sources)	Prolonged aPTT and PT; anticoagulant potency lower than heparin	ATIII-mediated FXa inhibition and direct thrombin inhibition
[90]	Sulfoethyl chitosan–Ag nanoparticles	Blood plasm +25 μL SECS@Ag–aPTT 62.5 s, PT 12.67 s; Blood plasm +50 μL SECS@Ag–aPTT 132.6 s, PT 12.78 s	Interference with FXa activity
[69,91,92]	Aryl-sulfonated chitosan derivatives	At different material concentrations, the aPTT increases from the control value (~33 s) to >250 s, PT increases from approximately 11–12 s (control) to about 17–26 s, and Anti-Xa activity is observed at around 0.09 UI/mL.	Activity on the extrinsic pathway was very low, and the factor Xa was completely inhibited.
[93,94,95]	Sulfated chitosan derivatives (various)	Moderate aPTT and TT prolongation; weaker than heparin	Heparin-like, ATIII-mediated thrombin inhibition
[73]	Low Molecular Weight Fucoidan (LMF)	Mild PT prolongation; moderate TT prolongation	FXII inhibition (intrinsic pathway)
[71]	Highly sulfated fucoidan derivatives	aPTT prolongation comparable to enoxaparin; no PT/TT effect	ATIII-independent inhibition of the intrinsic FXase complex
[94]	Fucoidan (*Holothuria polii*)	Lower aPTT/TT activity than heparin; IC_50_ ≈ 0.36 nM	HCII-mediated FIIa inhibition
[95]	Photodegraded fucoidan fractions (Dfuc1, Dfuc2)	*In vitro*: aPTT/TT prolongation; In vivo: prolongation of aPTT from 23.7 s (control) to Dfuc1 25.1 s, Dfuc2 29.4 s.	Molecular weight-dependent intrinsic pathway inhibition
[96]	Fucoidan (*Cystoseira myrica*)	aPTT prolonged at low doses; PT affected only at high concentrations	Predominantly intrinsic pathway modulation
[97]	Carboxymethyl κ-carrageenan multilayers	*In vitro* coagulation at a level similar to heparin systems	Surface-mediated anticoagulation
[98]	λ- and θ-carrageenan degraded by autohydrolysis	λ-carrageenan is more active than θ; both are weaker than heparin	ATIII-independent, surface-driven effects, molecular weight, and sulfation depend on
[99]	Chitosan–κ-carrageenan hydrogels	Strong aPTT and TT prolongation	Inhibition of intrinsic coagulation factors (FVIII, FIX, FXI, FXII)
[100]	Sulphated dextran, magnetic dextran sulphate	*In vitro*: aPTT > 250–300 s	Inhibition at the thrombin formation stage; reduced fibrin adsorption
[101,102]	Dextran sulfate derivatives	Prolonged PTT, TT, and PT *in vitro* and *in vivo*	Thrombin inhibition via HCII; modulation of the extrinsic pathway

**Table 3:** Review of crucial bioactive synthetic polymers with antithrombotic effect.

Refs.	Polymer	Synthesis/Coating Method	Antithrombotic Activity	Advantages/Disadvantages
[105,106,107]	Polyethylene glycol (PEG)	Surface PEGylation, crosslinked PEG hydrogels, and radical polymerization	*In vitro*: 80–95% reduction in fibrinogen adsorption; significant decrease in platelet activation; *In vivo*: prolonged bleeding time, reduced thrombogenicity of stents in animal models	Biocompatibility, hydrophilicity, strong antifouling effect/possible allergic reactions;
[121,122]	Poly(ethylene oxide) (PEO)	Block copolymerization, surface coatings	*In vitro*: low protein adsorption, high resistance to biofouling. *In vivo*: Improved patency of catheters; reduced thrombosis in animal models.	Nontoxicity, rapid clearance from the body, highly hydrophilic/high cost of functional derivatives
[108,123,124,125]	Poly(phosphorylcholine) (PMPC)	ATRP or RAFT polymerization, PMPC-based coatings	*In vitro*: near-complete suppression of fibrinogen adsorption; minimal platelet activation; *In vivo*: excellent hemocompatibility of vascular grafts; no thrombus formation in short-term studies	Biomimetic (cell membrane–like) behavior, excellent blood compatibility/complex synthesis, higher production cost
[113,126,127,128]	Fluoropolymers	Emulsion polymerization, fluoropolymer coatings	*In vitro*: reduced platelet adhesion and thrombin activation; *In vivo*: reduces thrombosis on occlusive implants	Strong antithrombotic properties/potential cytotoxicity of degradation fragments
[119,120]	Polyurethanes (PU) and their modifications	Incorporation of functional segments, solution casting	*In vitro*: inhibition of platelet activation and adhesion; *In vivo*: reduced early thrombosis in vascular prostheses	Good mechanical properties, good stability/risk of inflammatory response
[111]	Sulfonated block copolymers containing PEG segments	Radical or ionic polymerization	*In vitro* and *in vivo*: anticoagulant activity comparable to reference materials	Potentially safe therapeutic alternative for managing bleeding complications/limited clinical validation

**Table 4:** Overview of key polymers releasing antithrombotic factors.

Carrier/Material	Drug/Agent	Release Mechanism/Profile	Effects/Applications	Advantages/Limitations	Refs.
PLGA/PLA microspheres	Low Molecular Weight Heparin LMWH	Biphasic (burst + sustained phase); ester bond hydrolysis + polymer matrix degradation; PEG-assisted diffusion	Long-term release (14 days), anti-Xa activity maintained for ~6 days; *In vitro* studies using rabbit models. A promising direction for subcutaneous or intravascular antithrombotic drug delivery	Stable, scalable; burst release requires control.	[137]
PCL/PLGA /Eudragit nanoparticles	Heparin	Diffusion; stability in the GIT (digestive tract)	Prolonged anti-Xa activity; oral delivery (rabbit models)	Good pharmacokinetic; variable oral bioavailability	[146]
PLGA/PEG microspheres /nanoparticles	Apixaban/Rivaroxaban (inhibitory Xa)	Biphasic; PLGA hydrolysis + diffusion (PEG modifies kinetics) by 10–20 days	Synergy with heparin; improved bioavailability; *in vivo* rat/rabbit models; potential for therapy: AF-related stroke, prevention of venous thrombosis, long-term treatment, and local control of coagulation	Controlled release; stability	[147,151,153]
PLGA-SA; PS/hpGHA nanoparticles	Heparin (antyzakrzepowy)/glutathione (antyoksydant)	Biphasic; similar to PLGA-based systems	*In vitro* studies; ↑ APTT; reduced platelet activation; strong antioxidant effect; potential use in the treatment of vascular diseases	Multifunctional nanosystems; no *in vivo* data; risk of initial burst	[152]
Chitosan/TMC nanoparticles	Low Molecular Weight Heparin LMWH	Mucoadhesion + opening of tight junctions; pH stability	Increased oral LMWH bioavailability; ↑ APTT; *in vivo* rabbit model	Strong mucoadhesion; oral delivery; lack of long-term safety data	[154]
Self-assembled LMWH–octadecylamine nanoparticles	Low Molecular Weight Heparin LMWH heparina	Autocoagulation + diffusion from hydrated matrix	Activity duration 4–5 days; high anti-Xa activity, improving pharmacokinetics; *in vivo* rabbit models, a system mainly designed for systemic ogólnoustrojow	Good biocompatibility; effect still too short for implants; requires further research	[84]
Electrospun PCL fibers	Hepirain	Diffusion: very slow release	Inhibition of VSMC proliferation: potential for stent coatings	Slow degradation; no in vivo data	[155]
Responsive star-PEG–heparin hydrogel	Hepirain	Thrombin-cleavable linker → immediate release	APTT/TEG tests; reduced platelet adhesion; ex vivo circulation model, animal models (implanted coatings), reduction in surface clots	Smart system; complex synthesis; no clinical data	[140]
Pluronic F127 Triblock polymer (PEO-PPO-PEO) thermo-sensitive hydrogel	r-Hirudin (rHV2)	Dissolution-controlled; diffusion through gel	Depot effect; prolonged local activity (e.g., eye, subcutaneous); animal rabbit models	Biocompatible; suitable for local delivery; no responsiveness	[156]
Chitosan hydrogel	Unfractionated Heparin UFH/LMWH	(+NH_3_ ↔ –SO_3_^−^) + Ionic interactions + swelling	↑ APTT; ↑ anti-Xa; reduced platelet adhesion; future use as a compress	Simple preparation; biodegradable; limited mechanical stability	[139]
Layer-by-layer (LbL) coating	Heparin/antithrombin peptide	Ionic/pH-triggered desorption; multilayer breakdown; thickness-controlled kinetics	Prolonged clotting time; reduced platelet adhesion; tunable activity	Precise dosing; environmental sensitivity	[157,158]
Polyacrylamide nano-gels	r-Hirudin	Thrombin-accelerated release; protection from proteolysis	Local thrombin inhibition; reduced clot burden; mouse lung/artery models	Smart delivery; no long-term toxicity data	[159]
Microneedle patch— thrombin-responsive	Heparin—HA conjugate	Thrombin cleaves linker → instant “burst-on-demand” release	Autoregulated anticoagulation; non-invasive; *in vivo* studies: effective in mouse thrombosis models	Easy patient use; scaling challenges	[160]
PVP microneedle patch	r-Hiuridin	Diffusion + MN delivery	Prolonged inhibitor activity, decreased platelet activity 24 h release; 48 h biological effect; rat/porcine ear models; effectively alleviates the formation of congestion *in vivo*; application, long-term treatment of thrombotic diseases.	Minimally invasive; prolonged effect; limited mechanical strength	[161]
pH responsive/mucoadhesive thiolated chitosan(TCS) + (HPMCP) nanoparticle	Low Molecular Weight Heparin LMWH	pH-triggered release, mucoadhesion; disulfide bonding with mucin; transient TJ loosening	Strong epithelial penetration; gastric protection; oral delivery platform	High stability; no clinical data; risk of excessive permeation	[162]
PEG hydrogel + SNAP (NO + heparin)	Heparin + NO-donor	Diffusion + ROS-mediated NO generation	Anti-Xa + anti-biofilm; subcutaneous implants	Dual-function (NO + anticoagulant); NO instability	[163,164]
PC (Tyrocore III gen.)	Sirolimus	Degradation + diffusion	Improved endothelialization; no thrombosis; BRS in ST-segment elevation myocardial infarction (STEMI)	Proven clinical efficacy; limited industrial access	[138]

Where: ↑ APTT—increase of activated partial thromboplastin time (APTT), ↑ anti-Xa—increase in anti-Xa activity.

**Table 5:** Representative publications on bioactive polymers modified with anticoagulants, methods of their immobilization, mechanisms of action, and observed effects in *in vitro* and *in vivo* studies.

Polymer/Modification	Mechanism of Action	Effects/Applications	Refs.
Heparin–poly(ε-caprolactone) (PCL) conjugate	Heparin conjugation using EDC/NHS chemistry; The PCL–heparin conjugate is processed into nonwoven tubular scaffolds by electrospinning	*In vivo* studies in canine femoral artery grafts demonstrated good biocompatibility and patency of the implanted scaffolds compared with non-heparinized controls.	[172,173,174]
ATIII–heparin–PEG complex (catheter coating)	Complex of antithrombin III (ATIII), heparin, and PEG covalently bound to the catheter surface.	*In vivo*, ATH-coated catheters showed better patency than uncoated commercial catheters and those only heparin-coated	[175]
Polypropylene (PP) + heparin	PP surface activated by oxygen plasma etching, then functionalized with amine-rich plasma and finally covalently immobilized with heparin via EDC/NHS coupling.	*In vitro*: reduced hemolysis index and protein adsorption, decreased platelet adhesion, and strong reduction in bacterial adhesion (Gram-negative and Gram-positive strains). Dual antithrombotic and antibacterial functionality	[176]
Poly(tetrafluoro ethylene)(PTFE)+ PDA/PEI + heparin (ECM-like coating)	PTFE surface modified with a bioinspired coating based on polydopamine (PDA) and polyethyleneimine (PEI), followed by immobilization of heparin on the ECM-mimicking layer	Inhibits thrombus formation, prevents intimal hyperplasia, and promotes endothelialization on PTFE vascular grafts and related blood-contacting devices	[113]
Heparinized PLLA copolymer with controlled As_2_O_3_ release	Heparin covalently immobilized on the surface of a PLLA copolymer stent coating. As_2_O_3_ (arsenic trioxide) is incorporated as an antiproliferative drug; variant LA: MBC = 90:10 provides the slowest degradation	Temporal synergy: heparin protects against early thrombosis, As_2_O_3_ suppresses intermediate/late vascular smooth muscle cell proliferation and inflammation, while the degradable PLLA matrix enables controlled local dosing and reduces late hypersensitivity typical of durable polymers	[177,178]
Heparinized PLLA/PLCL tubular scaffolds	Heparin covalently coupled to PLLA/PLCL tubular scaffolds using EDC/NHS chemistry.	Heparinized scaffolds display increased surface hydrophilicity, reduced non-specific protein adsorption, and improved hemocompatibility; *in vivo* rabbit studies showed neovascularization within the scaffolds.	[170,179]
Bilayer grafts: outer PCL nanofibers, inner PLCL (50:50) sponge + heparin.	Bilayer design: outer layer of electrospun PCL nanofibers, inner sponge of PLCL (50:50) loaded with heparin; heparin conjugated via gas plasma treatment	*In vivo* adult sheep bilateral carotid interposition graft model: fast initial heparin release within 24 h followed by sustained low-level release over 14 days; strong inhibition of platelet adsorption and prevention of acute graft thrombosis	[171]
Catechol (CA)/polyethyleneimine (PEI) copolymer coatings + heparin	Coatings based on copolymerization of catechol (CA) and PEI on polymer substrates, followed by EDC-induced crosslinking to immobilize heparin onto the CA/PEI layer	Biomimetic, mussel-inspired coating; CA/PEI and CA/PEI–heparin coatings modulate endothelial cell behavior and provide antithrombotic surfaces for vascular grafts and stents	[180]
3D-printed PLA stent + PDA/PEI + heparin	3D-printed PLA stent first coated with PDA and PEI, creating a highly amine-rich surface; heparin is then immobilized via reaction of its carboxyl groups with surface amines (e.g., EDC/NHS)	Heparinized PLA stents fabricated under mild, aqueous, solvent-free conditions show markedly improved blood compatibility and antithrombotic activity *in vitro* and *in vivo*, with reduced neointimal hyperplasia.	[181]
PCL nanofibers + PDA + recombinant hirudin (rH)	Electrospun PCL nanofibers are functionalized by spontaneous oxidative polymerization of dopamine, forming a PDA layer; recombinant hirudin is then immobilized on PDA.	Strong thrombin inhibition with markedly prolonged clotting times; increased adhesion and proliferation of endothelial cells and decreased platelet adhesion; proposed for vascular tissue engineering (*in vitro* data).	[182]
PET (Dacron) + hirudin	PET vascular grafts modified by immobilizing hirudin on the surface using glutaraldehyde (GA) as a coupling reagent.	Compared with untreated PET, hirudin-modified PET showed reduced platelet and fibrinogen adhesion, improved antithrombotic properties, and inhibition of thrombin-induced vascular smooth muscle cell proliferation; *in vivo* canine implants confirmed improved graft performance.	[183,184]
PTFE with plasma activation + immobilized biomolecules	PTFE surface activated by plasma treatment, followed by chemical immobilization of bioactive molecules (e.g., heparin or direct thrombin inhibitors).	*In vitro*: improved hemocompatibility with reduced protein adsorption and platelet activation; potential for vascular grafts, catheters, stents, and other blood-contacting implants.	[185]
Silk fibroin (SF) films modified with hirudin	Silk fibroin films chemically modified by immobilization of hirudin on the SF backbone/surface.	Markedly improved antithrombotic properties of SF films; hirudin-modified SF supported adhesion and proliferation of endothelial and smooth muscle cells, with selective inhibition of smooth muscle cell proliferation at higher hirudin loadings.	[186]
316L stainless steel (SS) + PPAAm + bivalirudin (DTI)	316L SS surface coated with a thin PPAAm film rich in reactive amine groups; bivalirudin (direct thrombin inhibitor derived from hirudin) is covalently coupled to PPAAm	Significant prolongation of coagulation times and reduced platelet and fibrinogen adhesion/activation; *in vivo* canine models showed reduced thrombus formation due to rapid re-endothelialization.	[187]
PLA/PVP membrane with non-covalently bound natural hirudin	PLA membrane blended or coated with polyvinylpyrrolidone (PVP); natural hirudin is physically adsorbed and retained mainly through hydrogen bonding interactions (no covalent link).	*In vitro*, hirudin-containing PLA/PVP membranes significantly increased coagulation times vs. control, demonstrating improved hemocompatibility while avoiding chemical modification of the peptide.	[188]
PLA/PCL–gelatin vascular grafts with ACH11 and CAG peptides	Electrospun PLA/PCL/gelatin vascular grafts with co-immobilization of ACH11 antithrombotic peptide and CAG cell-adhesive peptide (covalent coupling onto the scaffold surface).	ACH11 inhibits the denaturation of adsorbed fibrinogen, suppresses FXa activation and platelet adhesion/aggregation; CAG selectively enhances endothelial cell adhesion, proliferation, and NO release. *In vivo*, ACH11/CAG-modified membranes showed improved patency and endothelialization.	[189]
PCU (polycarbonate urethane) with disulfide bridges—NO-releasing system	PCU modified with disulfide-containing segments that catalyze nitric oxide (NO) release from endogenous donors at the blood–material interface.	Decreased platelet adhesion, good compatibility with endothelial cells, inhibition of smooth muscle cell and macrophage proliferation, and significant antithrombotic effects *in vivo* (self-renewing, endothelium-mimicking NO-releasing surface).	[190]

**Table 6:** Classic examples of self-cleaning/antifouling coatings that reduce plate activation.

Coating Type	Mechanism	Main Hemocompatibility Effect	Refs.
Zwitterionic polymer coatings	Surface-grafted or bulk-modified poly(zwitterions) (e.g., sulfobetaine, carboxybetaine, phosphorylcholine) form a strongly hydrated, charge-neutral layer that sterically and electrostatically repels plasma proteins and cells.	Very low adsorption of fibrinogen and other plasma proteins, strong reduction in platelet adhesion/activation, and complement activation; improved hemocompatibility under whole blood and plasma flow.	[199,208,209,210,211]
Superhydrophobic/superhemophobic coatings (SHP)	Micro/nanostructured roughness combined with low-surface-energy chemistry (e.g., fluorinated silanes on titania nanotube arrays) creates a Cassie–Baxter state with trapped air, so blood contacts only a tiny fraction of the solid.	Markedly reduced protein (incl. fibrinogen) adsorption and platelet adhesion, decreased Factor XII activation, and delayed whole-blood clotting; surfaces behave as “blood-repellent.”	[206,212,213]
Phosphorylcholine (PC)-based coatings	Biomimetic MPC-containing copolymers tethered to device surfaces or bulk-functionalized poly(ester urethane) ureas; PC headgroups mimic phosphatidylcholine in cell membranes and maintain a tightly bound hydration shell that suppresses protein and cell binding.	Reduced thrombin generation, platelet and leukocyte activation, and complement activation during extracorporeal circulation; improved hemocompatibility of oxygenators, ECMO circuits, and degradable vascular scaffolds.	[197,201,202,207,214]
Self-cleaning/anti-fouling zwitterionic hydrogels		Strong suppression of fibrous capsule formation and chronic inflammation *in vivo*, low macrophage/giant-cell recruitment, reduced protein adsorption; improved long-term function of implanted gels and encapsulated cells (“stealth”, immune-evasive behavior).	[211,215,216]

**Table 7:** Comparison of the main heparin functionalization strategies.

	Adsorption (LbL)	Covalent Immobilization	Grafting To	Grafting From
Mechanism	Physical interactions: electrostatic, hydrophobic, protein-mediated complexes	Formation of chemical bonds (EDC/NHS, Schiff base, epoxy coupling)	Attachment of pre-activated heparin molecules to reactive surface groups	Polymerization initiated directly from heparin (SI-ATRP, RAFT)
Preservation of the heparin conformation	Very good	Good–moderate (dependent on crosslink density)	Very good (minimal modification of the native structure)	Very good
Stability under flow conditions	Low–moderate	High	High	Very high
Packing density of the surface layer	Limited; governed by physical interactions	Moderate–high	Moderate (limited by macromolecule diffusion)	Very high (“polymer brush”)
Architectural precision of the surface	Moderate	Moderate	High	Very high
Heparin bioactivity	Highest (no structural modification)	Method-dependent (EPA > MPA)	Very high	Very high
Synthetic difficulty	Low	Moderate	Moderate	High
Cost	Lowest	Moderate	Moderate	High
Optimal applications	Low-cost coatings, wound dressings, growth-factor release systems	Vascular grafts, implants, and long-term stable coatings	Bioactive coatings requiring preserved heparin activity	Advanced brush-type coatings, responsive interfaces, and intelligent biomaterials
Main limitations	Leaching, protein interference	Risk of the chain rigidification	Limited surface density	High cost, greater chemical complexity

**Table 8:** Summary of Representative Studies on PEG–Heparin Functionalization.

System	Type of Functionalization	Application	Ref.
Electrospun tubular scaffolds made of PEUU	Covalent grafting of PEG and heparin	Development of vascular grafts with high conformability and rapid endothelialization	[243]
Pegylated heparin-based nanopolymers: HP-Ppa-mPEG and redox-responsive HP-Ppa-SS-mPEG	Chemical pegylation of heparin (stable vs. GSH-responsive linkers)	Smart nanocarriers for photodynamic therapy (PDT)	[244]
PEGDA/PEGMA hydrogels with incorporated heparin; coatings on PMP membranes (ECMO)	Heparin is physically incorporated into the PEG-based hydrogel matrix	Antithrombotic coatings for ECMO membranes	[234]
Star-shaped PEG hydrogels with maleimide-functionalized heparin	Heparin is integrated into a hydrogel network	Long-term *ex vivo* model for human breast tissue culture	[245]
6-arm PEG–heparin copolymer immobilized on chitosan-modified magnesium alloy	Electrostatic/adsorptive immobilization of PEG-Hep	Surface modification of biodegradable Mg stents to enhance corrosion resistance and hemocompatibility	[235]
StarPEG–heparin hydrogel used as a biosensing matrix	Heparin embedded in star-shaped PEG hydrogel	Biosensors for IgG detection	[246]
Heparin-b-PEG conjugate (oxime ligation)	Covalent pegylation of heparin (end-on)	Modifying heparin to prolong half-life and reduce side effects	[238]

**Table 9:** Comparison of the main polymer classes of heparin-mimetics.

	Mechanism of Action (How It Mimics Heparin)	Advantages	Disadvantages	Typical Applications	Refs.
Synthetic sulfonated polymers (PSSS, PSPMA)	Anionic –SO_3_^−^ groups imitate the “charge cloud” of heparin; stabilization of BMP, FGF, VEGF	High stability, easy control of MW, and architecture, excellent reproducibility	Lack of natural sequence-specific recognition motifs (no HS fine structure)	Tissue engineering, growth factor stabilization, vascular biomaterials	[239,249]
RAFT-synthesized heparin-mimetic block copolymers	Precise positioning of sulfonate groups; strong stabilization of FGF-2	Structural uniformity, excellent control over block length	Complex synthesis requires CTA and optimization. polymerization conditions	Pro-angiogenic systems, advanced scaffolds	[250]
Zwitterionic sulfonated polymers (SBMA, PSBMA)	Synergy of zwitterionic and anionic behavior; selective binding of FGF-2	Outstanding anti-fouling properties + strong retention of growth factors	More difficult surface conjugation; possible hydrogel stiffening	Skin regeneration, angiogenesis, pro-regenerative hydrogels	[252,253]
Synthetic glycopolymers (HS-mimicking)	Precise positioning of sulfate motifs; specific GAG–protein interactions	Highest structural control; ability to reproduce HS binding sequences	Highly complex synthesis; high cost	Heparanase inhibitors, antiviral materials, biosensors, and ECM regulation	[254,255]
Sulfonated natural polysaccharides (S-HA, S-chitosan)	Sulfonation generates a charge density. similar to heparin	Biodegradable, biocompatible, easy to functionalize	Lower structural specificity compared to native heparin	TGF-β stabilization, chondrogenesis, and anti-metastatic systems, wound dressings	[247,256,262]
Synthetic sulfonated polypeptides/polyesters	Controlled placement of –SO_3_^−^ groups on a rigid or soft polymer chain	Very high predictability, no animal-derived antigens	Emerging technology—limited clinical data	Coagulation regulation, ROS-responsive materials, and antibacterial materials	[259,260]
Sulfonated polyglycerols and dendritic networks	Multi-arm architecture increases local charge density	Very high protein retention, excellent solubility, strong stability	Possible difficulty in controlling arm sulfonation	Nanodrug carriers, hydrogels, bioactive coatings	[261]

**Table 10:** The standardized tests for evaluating hemocompatibility and thrombogenicity of polymers.

Type of Test	Purpose/Scope	Method/Parameters	Typical Indicators	Standards/Ref.
Clotting time	Assessment of the activation of the coagulation cascade by the material	PT (Prothrombin Time)—Extrinsic + common coagulation pathways, aPTT (Activated Partial Thromboplastin Time)—Intrinsic + common pathways (factors XII, XI, IX, VIII, X, II, I), TT (Thrombin Time)—fibrin formation	Change in clotting time compared to control	ISO 10993-4:2017 [265]
Fibrinogen/Fibrin determination	Evaluation of fibrin formation after blood contact with the material	Measurement of fibrinogen concentration in plasma, and microscopic observation of clot formation	Fibrinogen depletion, presence of fibrin fibers	ISO 10993-4:2017 [265]
Platelet activation	Investigation of material influence on platelet activation and adhesion	Optical or impedance aggregometry, flow cytometry (CD62P, CD63), SEM imaging	% of activated platelets, number of adhered platelets, expression of activation markers	ISO 10993-4:2017 [265], ASTM F2888-13 [88]
Hemolysis test	Evaluation of red blood cell damage upon contact with the material	Incubation of the sample with blood, measurement of free hemoglobin (e.g., spectrophotometrically)	% hemolysis (0–2%: non-hemolytic, 2–5%: slightly hemolytic, >5%: hemolytic)	ASTM F756-17 [266], ISO 10993-4:2017 [265]
Complement activation	Assessment of the immunological reactivity of the material surface	Measurement of C3a, C5a, SC5b-9 by ELISA	Increased concentration of complement activation proteins	ISO 10993-4:2017 [265]
Protein adsorption	Determination of the quantity and type of plasma proteins adsorbed on the surface	Spectroscopic analysis (FTIR, CD), SDS-PAGE, and immunochemical assays	Amount of adsorbed fibrinogen, albumin, etc.	ISO 10993-4:2017 [265]
Dynamic flow tests	Simulation of blood flow conditions in vascular systems	Closed-loop flow system, measurement of cell morphology, aggregation, and clot formation	Cell morphology, clot mass, and number of activated platelets	ASTM F2888-13 [268], ASTM F2150-19 [267]
Surface morphology after blood contact	Visualization of surface changes after exposure to blood	SEM, optical, or confocal microscopy	Presence of platelets, fibrin, erythrocytes	ISO 10993-4:2017 [265]

**Table 11:** *In vivo* models for assessing the thrombogenic properties of polymers.

Model Type	*In Vivo* Thrombogenicity Model	Animal Model	Typical Application	Advantages	Key Assessment Methods	Refs.
Short-term	Arteriovenous (AV) Shunt	Rabbit, Rat, Pig, Dog	Dynamic evaluation of blood-material interaction under flow	Physiologically relevant flow, sensitive to platelet/fibrin deposition	Platelet/fibrin quantification, SEM, blood coagulation markers	[257,279]
	Extracorporeal Circulation (ECC)/Loop	Pig, Dog, Hamster, Sheep	Testing catheters, tubing, and vascular grafts	Clinically relevant flow, suitable for larger devices	Thrombus weight, platelet adhesion, coagulation markers, and imaging	[81,267,280]
Usually short-term, rarely long-term	Catheter Implantation	Rabbit, Rat	Assessment of intravascular devices	A simple surgical procedure allows repeated sampling	Occlusion time, platelet counts, histopathological analysis, SEM, and thrombus scoring	[128,281]
Long-term	Vascular Graft Implantation	Sheep, Pig, Rat, Rabbit	Evaluation of polymeric grafts	Physiologically relevant vessel size, long-term endothelialization study	Histological evaluation, endothelialization assessment, thrombus quantification, angiography, SEM	[282,283,284]
	Stent Implantation	Pig, Rabbit	Thrombogenicity and endothelialization of stents	High translational relevance	Angiography, SEM, histopathological analysis, platelet/fibrin deposition	[285,286,287]
	Small Vessel Implantation (e.g., discs, femoral artery/vein)	Rat, Rabbit	Screening of polymer coatings or small devices	Low cost, short experimental duration	Histology, platelet adhesion assays, fibrin deposition, SEM	[81,283,288]

**Table 12:** Clinical and translational applications of antitrombotic/antifouling coatings.

Device Category	Example Product/Technology	Type of Coating/Surface Treatment	Clinical/Translational Application & Status	Refs.
Peripheral vascular grafts	GORE^®^ PROPATEN^®^ Vascular Graft (ePTFE) (GORE, Phoenix, AZ, USA)	CBAS^®^ covalently bound heparin layer on luminal ePTFE surface	Routine clinical use for lower-extremity bypass and AV access; improved patency and reduced early thrombosis vs. standard ePTFE grafts	[338,339,340]
AV access grafts for hemodialysis.	Flixene^®^ Vascular Graft (Getinge, Göteborg, Sweden)	Modified ePTFE with tailored porosity and surface for “early access.”	Clinical use as early-access AV graft; can be cannulated within 24–72 h (up to 7 days), reducing the need for temporary central venous catheters.	[325,341]
Coronary stents	COBRA PzF™ NanoCoated Coronary Stent (Symplr, Houston, TX, USA)	Ultrathin CoCr stent with ≤50 nm Polyzene-F^®^ fluoropolymer nanocoating	PCI, especially in high-bleeding-risk patients, with high thromboresistance and rapid endothelial coverage, enabling studies of very short DAPT regimens	[303]
Neurovascular flow diverters	FRED X Flow Diverter with “X Technology” (Terumo Neuro, Tokyo, Japan)	Antithrombotic polymer surface (PMEA-based “X Technology”) on nitinol flow diverter	Clinical treatment of intracranial aneurysms; multicentre data show low thromboembolic complication rates and high effective occlusion rates	[326]
Central venous catheters	Heparin-bonded CVCs (e.g., ICU multi-lumen catheters) (ICU Medical, Inc. San Clemente, CA, USA)	Heparin is covalently or ionically bound to the polyurethane catheter surface.	Routine clinical use in adults and children; RCTs and meta-analyses show reduced catheter-related thrombosis and improved patency vs. uncoated CVCs	[342]
Central venous/IV catheters—next-generation	Heparin-network-mediated long-lasting coating (experimental)	Cross-linked heparin network on the catheter surface, designed for a very durable antithrombotic effect	Preclinical/early translational stage; prolonged resistance to thrombosis and biofouling in animal models; intended for long-term CVC use (oncology, cardiology)	[304]
Hemodialysis blood lines & dialyzers	Heparin-coated hemodialysis circuits(heparin-coated membranes (np. AN69ST))	Heparin-bonded inner surfaces of tubing and dialyzer housing	Clinical use in selected centres to reduce clot formation in the extracorporeal circuit and allow lower systemic heparin doses in high-bleeding-risk patients.	[331]
ECMO circuits & centrifugal blood pumps	Heparin-coated ECMO circuits (e.g., Bioline^®^-type coatings) (MAQUET Cardiopulmonary AG Hirrlingen, Germany)	Systemic heparin bonding on oxygenator, tubing, and pump surfaces	Widely used for ECMO; reduces activation of coagulation and complement, lowers in-circuit thrombosis, and may permit less aggressive systemic anticoagulation.	[332,333]
Cardiopulmonary bypass (CPB) systems	PMEA-coated oxygenators and CPB circuits	Poly(2-methoxyethyl acrylate) (PMEA) hydrophilic polymer on blood-contacting surfaces	Routine use in modern CPB systems decreases platelet and complement activation; a model for similar antithrombotic/antifouling strategies in other devices.	[334]

## Data Availability

No new data were created or analyzed in this study. Data sharing is not applicable to this article.
